# Environmental genomics of Late Pleistocene black bears and giant short-faced bears

**DOI:** 10.1016/j.cub.2021.04.027

**Published:** 2021-04-19

**Authors:** Mikkel Winther Pedersen, Bianca De Sanctis, Nedda F. Saremi, Martin Sikora, Emily E. Puckett, Zhenquan Gu, Katherine L. Moon, Joshua D. Kapp, Lasse Vinner, Zaruhi Vardanyan, Ciprian F. Ardelean, Joaquin Arroyo-Cabrales, James A. Cahill, Peter D. Heintzman, Grant Zazula, Ross D.E. MacPhee, Beth Shapiro, Richard Durbin, Eske Willerslev

**Affiliations:** 1 Lundbeck Foundation GeoGenetics Centre, GLOBE Institute, University of Copenhagen, Copenhagen, Denmark; 2 Department of Zoology, University of Cambridge, Downing Street, Cambridge CB2 3EJ, UK; 3 Department of Genetics, University of Cambridge, Downing Street, Cambridge CB2 3EJ, UK; 4 Department of Biomolecular Engineering and Bioinformatics, University of California, Santa Cruz, Santa Cruz, CA, USA; 5 Department of Biological Sciences, University of Memphis, 3770 Walker Avenue, Ellington Hall, Memphis, TN 38152, USA; 6 State Key Laboratory of Tibetan Plateau Earth System Science (LATPES), Beijing 100101, China; 7 Department of Ecology and Evolutionary Biology, University of California, Santa Cruz, Santa Cruz, CA, USA; 8 Unidad Académica de Antropología, Universidad Autónoma de Zacatecas, Campus II, Col. Progreso, 98066 Zacatecas, Mexico; 9 The Archaeology Centre, Department of Anthropology, University of Toronto, 19 Ursula Franklin Street, Toronto, ON M5S 2S2, Canada; 10 Laboratorio de Arqueozoologia, Subdireccion de Laboratorios y Apoyo Academico, Instituto Nacional de Antropologia e Historia, Moneda 16, Col. Centro, 06060 Mexico, CdMx, Mexico; 11 Laboratory of the Neurogenetics of Language, Rockefeller University, New York, NY, USA; 12 The Arctic University Museum of Norway, UiT - The Arctic University of Norway, PO Box 6050, Langnes, N-9037 Tromsø, Norway; 13 Yukon Palaeontology Program, Department of Tourism & Culture, Government of Yukon, Whitehorse, YT Y1A 2C6, Canada; 14 Department of Mammalogy, American Museum of Natural History, New York, NY 12484, USA; 15 American Museum of Natural History, New York, NY, USA; 16 Howard Hughes Medical Institute, University of California, Santa Cruz, Santa Cruz, CA, USA; 17 Wellcome Sanger Institute, Cambridge CB10 1SA, UK; 18 MARUM, University of Bremen, Bremen, Germany

## Abstract

Analysis of ancient environmental DNA (eDNA) has revolutionized our ability to describe biological communities in space and time,^1–3^ by allowing for parallel sequencing of DNA from all trophic levels.^4–8^ However, because environmental samples contain sparse and fragmented data from multiple individuals, and often contain closely related species,^9^ the field of ancient eDNA has so far been limited to organellar genomes in its contribution to population and phylogenetic studies.^5,6,10,11^ This is in contrast to data from fossils^12,13^ where full-genome studies are routine, despite these being rare and their destruction for sequencing undesirable.^14–16^ Here, we report the retrieval of three low-coverage (0.03×) environmental genomes from American black bear (*Ursus americanus*) and a 0.04× environmental genome of the extinct giant short-faced bear (*Arctodus simus*) from cave sediment samples from northern Mexico dated to 16–14 thousand calibrated years before present (cal kyr BP), which we contextualize with a new high-coverage (26×) and two lower-coverage giant short-faced bear genomes obtained from fossils recovered from Yukon Territory, Canada, which date to ~ 22–50 cal kyr BP. We show that the Late Pleistocene black bear population in Mexico is ancestrally related to the present-day Eastern American black bear population, and that the extinct giant short-faced bears present in Mexico were deeply divergent from the earlier Beringian population. Our findings demonstrate the ability to separately analyze genomic-scale DNA sequences of closely related species co-preserved in environmental samples, which brings the use of ancient eDNA into the era of population genomics and phylogenetics.

## Graphical abstract

**Graphical abstract:**
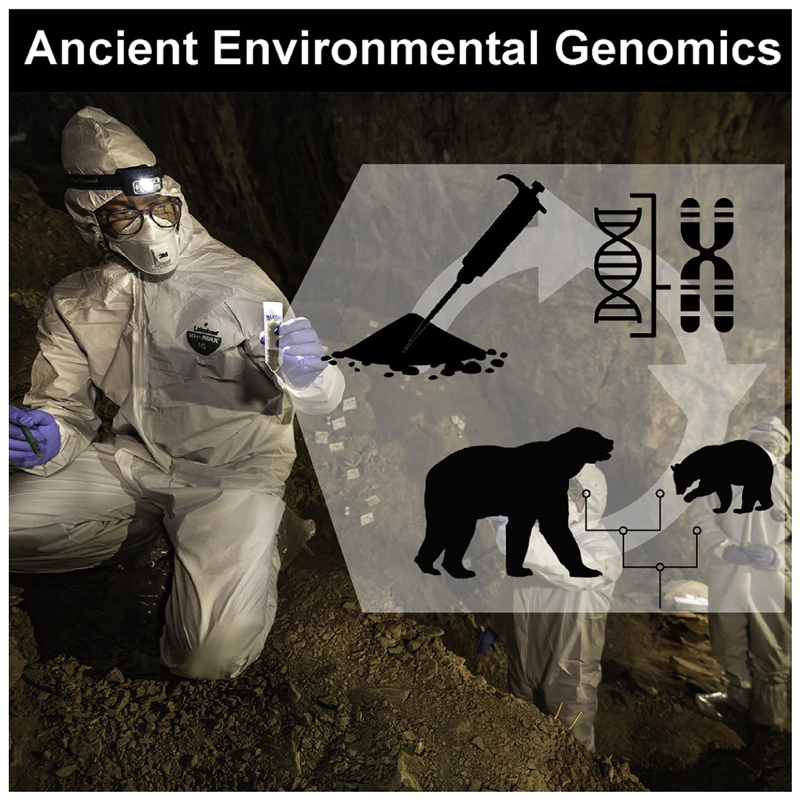

## Results and Discussion

Fossil records are incomplete, and many mammalian taxa, in particular those that lived at low population densities, are seldom found. For these rare taxa, destructive DNA extraction from fossil remains has the potential to reveal new insights into population and evolutionary history; however, it also causes irreversible damage to high-value specimens.^14–16^ The from past populations of organisms could be obtained directly from sediment, therefore, held great promise for ancient population genetics and phylogenetics.^1^ However, significant challenges of recovering and analyzing ancient DNA from sediments and disambiguating closely related species from mixed ancient DNA extracts have thus far held the field back from achieving this promise.

Commonly known as environmental DNA, or eDNA, research,^17^ this approach relies on sequencing DNA fragments derived from shed cells, hair, feces, and urine^18^ preserved within sediment. Standard eDNA techniques allow species composition to be determined in the absence of macrofossils across a variety of environments including sediments, speleothems, ice cores, lakes, rivers, and oceans.^19,20^ To date, however, analyses of ancient eDNA have been restricted to organellar DNA (mitochondrial and chloroplast) or, more recently, to short and highly diverse sequences generated using a “shotgun sequencing” approach,^4–6,8,10,11,20,21^ and ancient eDNA research has been restricted largely to simple taxonomic profiling of biological communities rather than population genetic or phylogenetic studies.

Here, we investigate whether it is feasible to retrieve genome-wide data directly from ancient environmental DNA. We obtained cave sediment samples from Chiquihuite Cave, Astillero Mountains, North Mexico, that were screened previously for the presence of American black bear (*Ursus americanus*) DNA^22^ and selected three strata in which black bear DNA was present for further processing. The first two strata, UE1210 and UE1212, have been dated to ~16–15 thousand calibrated years before present (cal kyr BP) by Ardelean et al.,^22^ after the peak of the last glacial maximum (LGM) but prior to the onset of Holocene warming at ~12.0 cal kyr BP, and radiocarbon dates from three charcoals (this study) place UE1605 between 15.0 and 13.0 cal kyr BP.

We recovered DNA from a total of 48 sediment subsamples within the three stratigraphic layers (Table S1). We converted extracted ancient DNA (aDNA) from these samples into 65 libraries for Illumina shotgun sequencing (STAR Methods). Competitive mapping against the RefSeq mitochondrial database (version 92) confirmed the presence of American black bear DNA across all three sedimentary layers (Figure S1A). Reads with the least edit difference to both the black bear mitochondrial genome and whole genome (assembly ASM334442v1) had elevated rates of deamination at fragment termini, as is characteristic for ancient DNA (Figures S1 and S2E–S2G).

### The Mexican black bear

**Figure 1:**
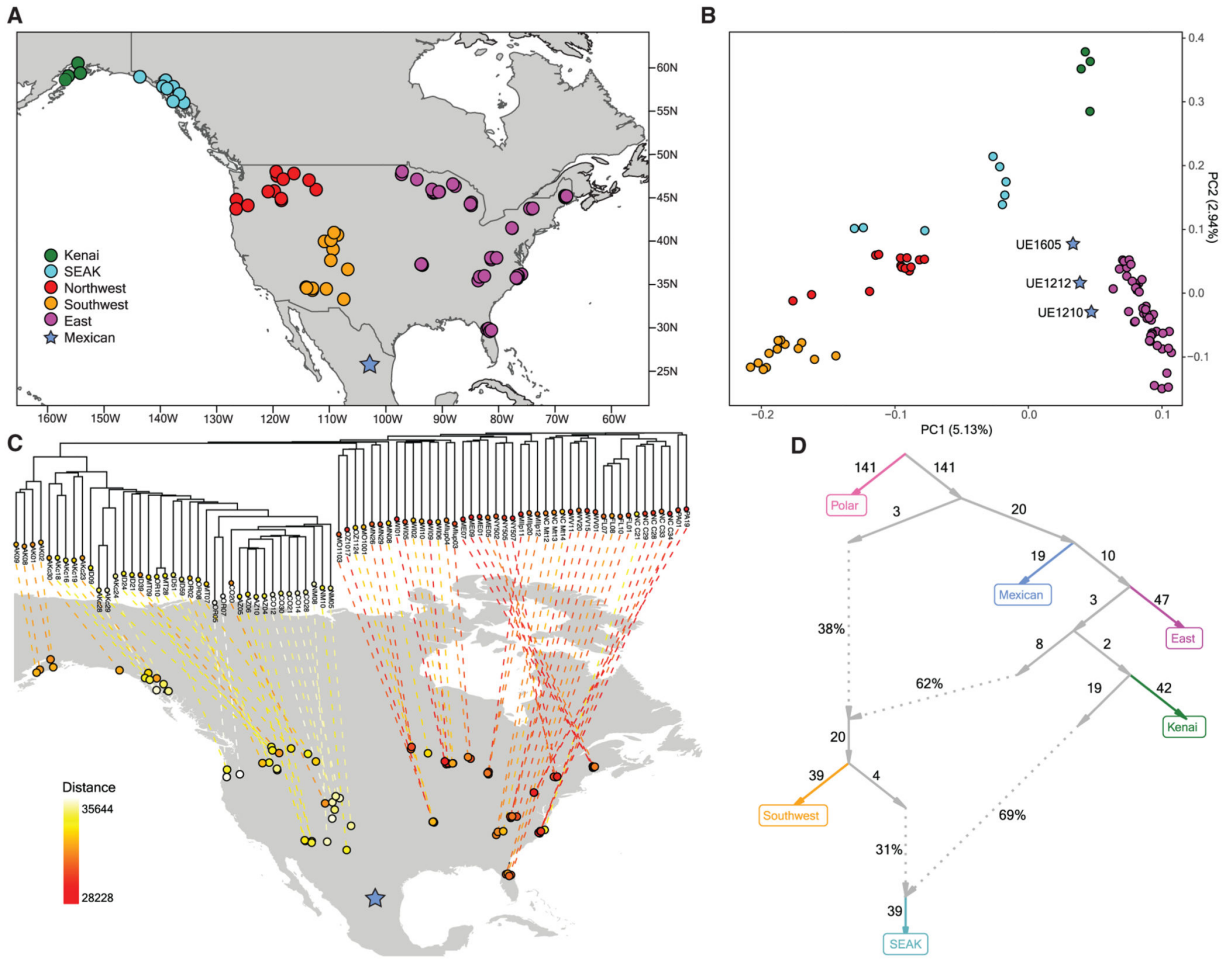
American black bear phylogeny (A) Map showing the black bear samples used. (B) Principal component analysis using *smartpca*, which accounts for the high amount of missing data in the Mexican samples by projecting the ancient samples onto a PCA created from the modern samples. (C) Genetic Hamming distance of UE1212 to each of the modern samples on biallelic SNPs, scaled to account for missing data, mapped to a color scale, and plotted on a *phylomap* using a neighbor-joining tree of the modern samples (results for UE1210 and UE1605 are shown in Figures S3A and S3B). (D) Inferred admixture graph, using two polar bear genomes (STAR Methods) as an outgroup in our admixture analysis. All data were parsed and plotted using *admixtools2*. We determined seven best-fitting graphs with highly similar topologies and many shared characteristics. The best of these is shown here, with a score of 4.922, and with a worst excess f4 residual of −2.182 for the configuration (East,Kenai;Mexican,Polar), and the rest are shown in Figure S4. See also Figures S1–S4.

Using a panel of 83 present-day American black bears, we found that the black bear environmental genomes recovered from the three Mexican sediment layers are closely related to modern black bears from eastern North America, but also share ancestry with bears in present-day Alaska. Based on a combination of genetic data and topological features likely to impede gene flow, we assigned genomic data from 83 modern black bears^23^ to 5 geographically distinct populations in the United States: Kenai Peninsula (Alaska), Southeast Alaska (SEAK), Northwest, South-west, and East (Figure 1A). We then projected the three ancient eDNA samples into a principal component analysis (PCA) of the modern black bears using *smartpca*^24^ (Figure 1B). All three ancient samples clustered together and closest to the present-day East population. We next estimated a neighbor-joining tree of the modern samples (Figure 1C). We colored the modern samples in a *phylomap*^25^ according to their genetic Hamming distance from each of the ancient Mexican samples, which we rescaled using Plink to account for missing data. As in the PCA, the ancient Mexican black bears clustered most closely to the East population (UE1212, Figure 1C; UE1210 and UE1605, Figure S3), and closer to both Alaskan populations (Kenai and SEAK), than to the North-west and Southwest populations.

Admixture analysis revealed that the eastern lineage, to which we find that the Mexican bears belong, was the earliest to diverge from other present-day populations of American black bears (Figure 1D). We used *admixtools*2^26^ to obtain an admixture graph using the three Mexican black bears; the modern East, Southwest, SEAK, and Kenai populations; and two polar bears (*Ursus maritimus*) for an outgroup. This indicates that the ancient Mexican population diverged from the ancestral East population after the initial divergence between the eastern and western lineages of black bears. Divergence of the eastern lineage continued into branches that produced most Alaskan ancestry. Further, this diverged eastern lineage admixed with the western lineage in an ancestral population to the modern Southwest. A second admixture event occurred with a western population to produce the modern SEAK population.

**Figure 2:**
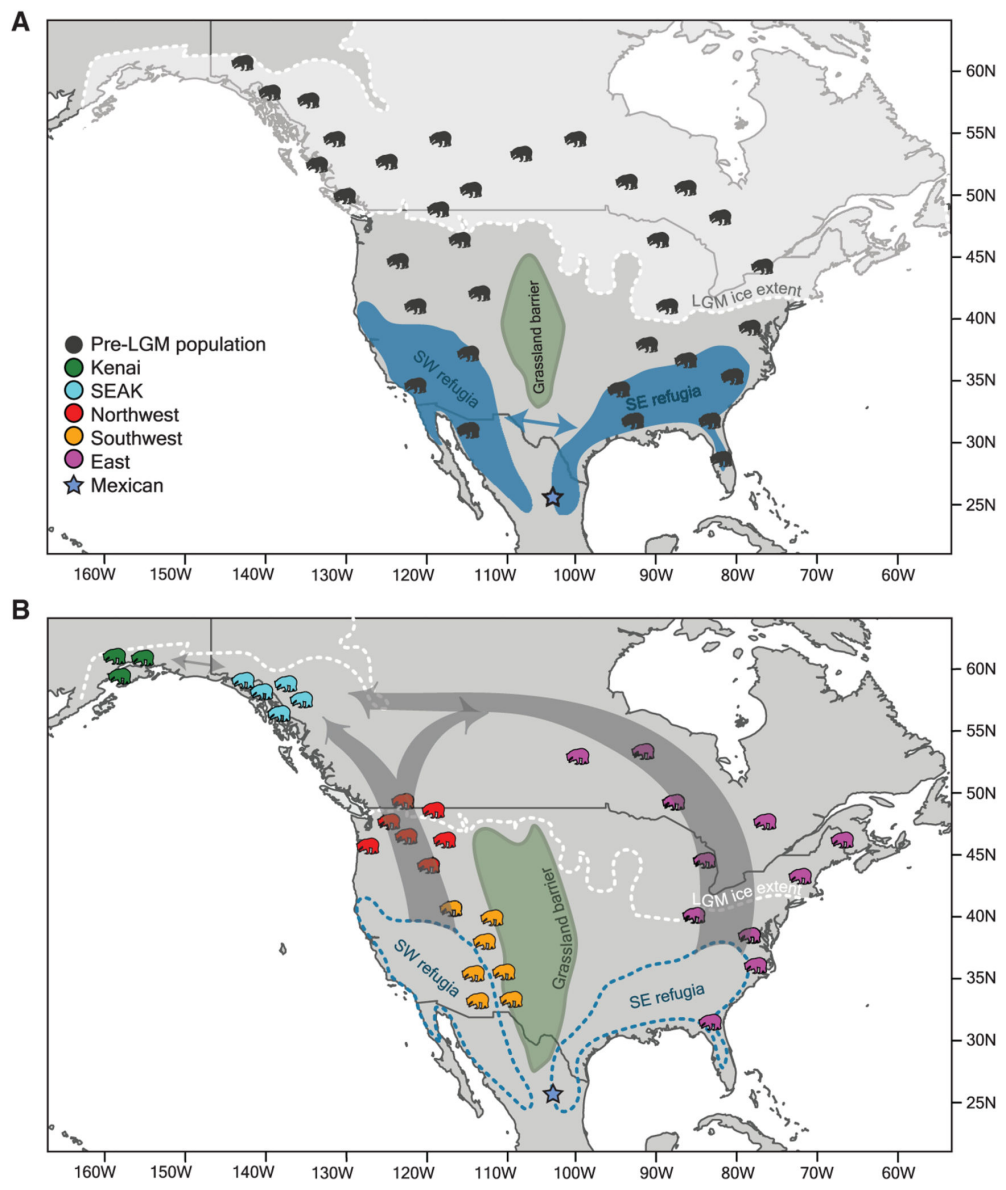
Working model of American black bear phylogeography (A) Pre-LGM–LGM conditions, with the ice sheet extending at ~21.5 kyr BP, and the hypothesized refugia to which the pre-LGM black bear population was suppressed. (B) Post-LGM conditions, with gray arrows indicating the northward recolonization of ice-free areas. See also Figures S3 and S4 and Table S2.

Our results expand and refine the working model of American black bear phylogeography, with our main hypothesis shown in Figure 2. Black bears first appeared in North America in the Late Pliocene,^27,28^ where they live today as forest generalists able to utilize resources from diverse forest compositions ranging from subtropical to boreal. Previous work reported that American black bears cluster into two major lineages in the eastern and western parts of the continent, and estimated that these lineages diverged ~67 cal kyr BP, possibly becoming separated by expanding grasslands across the central continent.^23^ However, genomic similarities between black bears in the East and those living in the most northerly population in Alaska^23^ suggest that the lineages may have remained connected during the Late Pleistocene, perhaps by forest habitats that spanned latitudinally across the northern continent, as they are today.^29^ Our population admixture graph supports this hypothesis, as we observed a lineage diverge from the East that constitutes the Kenai population and contributes a large portion of genetic ancestry to SEAK following admixture from western lineage populations (Figure 1D). Our inferred earlier divergence between the Mexican population and the population ancestral to both the East and Alaskan populations (Figure 1D) suggests either that there may have been two waves of colonization of the eastern range or, alternatively, that the East and Alaskan populations are descendants of a northward range expansion from a southern population. Our PCA (Figure 1B) shows a signature of range expansion in the east, which may be explained in two, non-mutually exclusive ways. First, range expansion into the eastern mountain ranges may have begun in the north and proceeded southward, resulting in isolation-by-distance or population structure. When glaciers advanced toward the peak of the ice age, northern bear populations contracted southward into the Southeast refugium (Figure 2A), where they maintained geographically structured populations rather than mixing with established bears. In this case, the leading edge of the northward expansion after the peak of the ice age would have comprised the descendants of the northern populations. Alternatively, northern populations may have been extirpated (or panmixia occurred), and the range expansion signal reflects expansion of the refugial population during post-glacial reforestation. The substructuring in the East may also be influenced by more recent processes: specifically, admixture from the North-west into populations around the Great Lakes^23^ (Figure 2B), which has resulted in higher diversity (unpublished data).

Contemporary Mexico has isolated bear populations in both the Sierra Madre Occidental and Sierra Madre Oriental mountain ranges, and is the only range state where black bears are considered endangered. Assuming population continuity in Mexico over the past 16 cal kyr BP, our results provide the first direct evidence linking eastern Mexican populations to the eastern lineage. Mitochondrial haplotype analyses identified clades A-west and A-east, respectively, in the Occidental and Oriental ranges,^30–32^ yet mitochondrial-nuclear discordance has been observed between bear species and in black bear populations. Combined, the data suggest two colonizations of the Mexican mountain ranges by black bears, and that the Chihuahuan Desert may have been a barrier to east-west gene flow. We show that the ancient Mexican population diverged before the East and Alaskan populations split; thus, given previous population divergence times from nuclear genomic data, we infer the Mexican population diverged 67–31 cal kyr BP.^23^

### The Mexican giant short-faced bear

**Figure 3:**
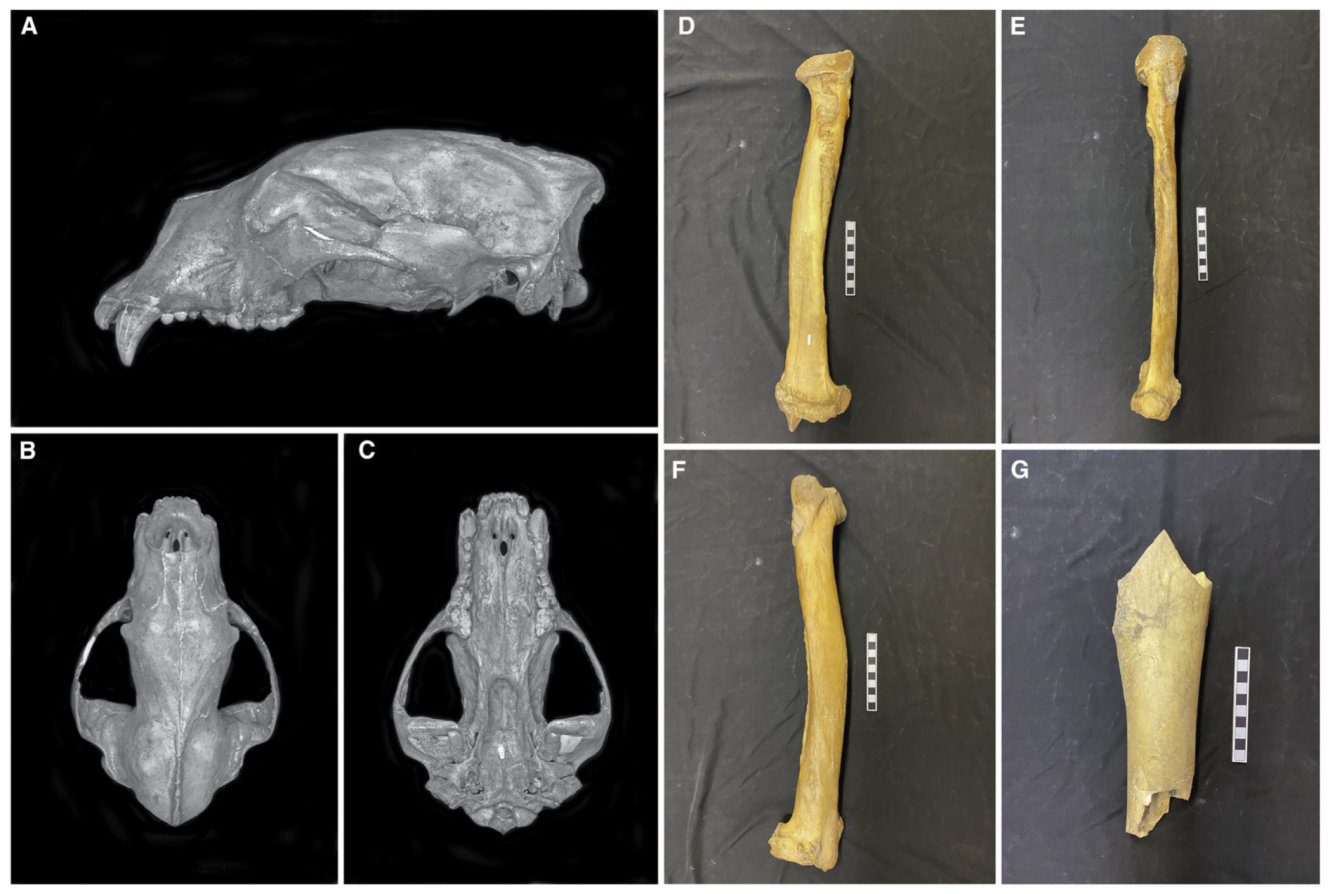
Photographs and descriptions of the three specimens used to generate the giant short-faced bear (*Arctodus simus*) genomes (A–C) YG 24.1, a complete cranium of a giant short-faced bear (*A. simus*) that was collected from Pleistocene age permafrost sediments exposed at a placer gold mine along Ophir Creek, Yukon Territory, Canada. All measurements on the cranium demonstrate this is a very small individual compared to other specimens of this species that have been described from the region.^33^ All sutures appear to be fused and all adult teeth are present and fully erupted, demonstrating this individual was an adult. As a high degree of sexual dimorphism has been demonstrated for *A. simus*, it is likely this small cranium represents an adult female. (D–F) YG 76.4, a complete radius bone from a giant short-faced bear (*A. simus*) that was collected from Pleistocene age permafrost sediments exposed at a placer gold mine along Hester Creek, Yukon Territory, Canada. The very large size of this radius precludes it from being any other large Pleistocene carnivore known from the region. The bone exhibits a high degree of bone exostosis on major muscle attachments, suggesting this represents an older adult male. This radius (YG 76.4) articulates with specimen YG 129.1, a complete right ulna, which was collected at the same locality. (G) YG 546.562, a small fragment of a right femur diaphysis collected from Pleistocene age permafrost sediments exposed at a placer gold mine along Canyon Creek, Yukon Territory, Canada. The thick cortical bone wall and curvature of the diaphysis clearly compare well with those of a giant short-faced bear (*A. simus*). See Table S3 for measurements and radiocarbon ages. See also Figures S1 and S2 and Data S1.

Exploratory analyses revealed that stratum UE1605 contained what appeared to be a mixture of DNA from two bear species (Figure S1A). When mapping reads recovered from this layer, some reads better aligned to the mitogenome of the giant short-faced bear (*Arctodus simus*) than to the reference genome of the black bear, with both showing an equal edit distance and high amount of DNA damage (Figures S1 and S2D). To explore this further, we extracted ancient DNA from three Late Pleistocene short-faced bear fossils that were recovered from perma-frost sediments in the Klondike goldfields of Yukon Territory, Canada (YG 24.1 [50.0 cal kyr BP], YG 76.4 [28.9 cal kyr BP], and YG 546.562 [29.8 cal kyr BP]) (Figure 3). We assembled complete mitochondrial genomes and nuclear genomic datasets from all three, including a 26-fold coverage nuclear genome for YG 564.562 (STAR Methods).

**Figure 4:**
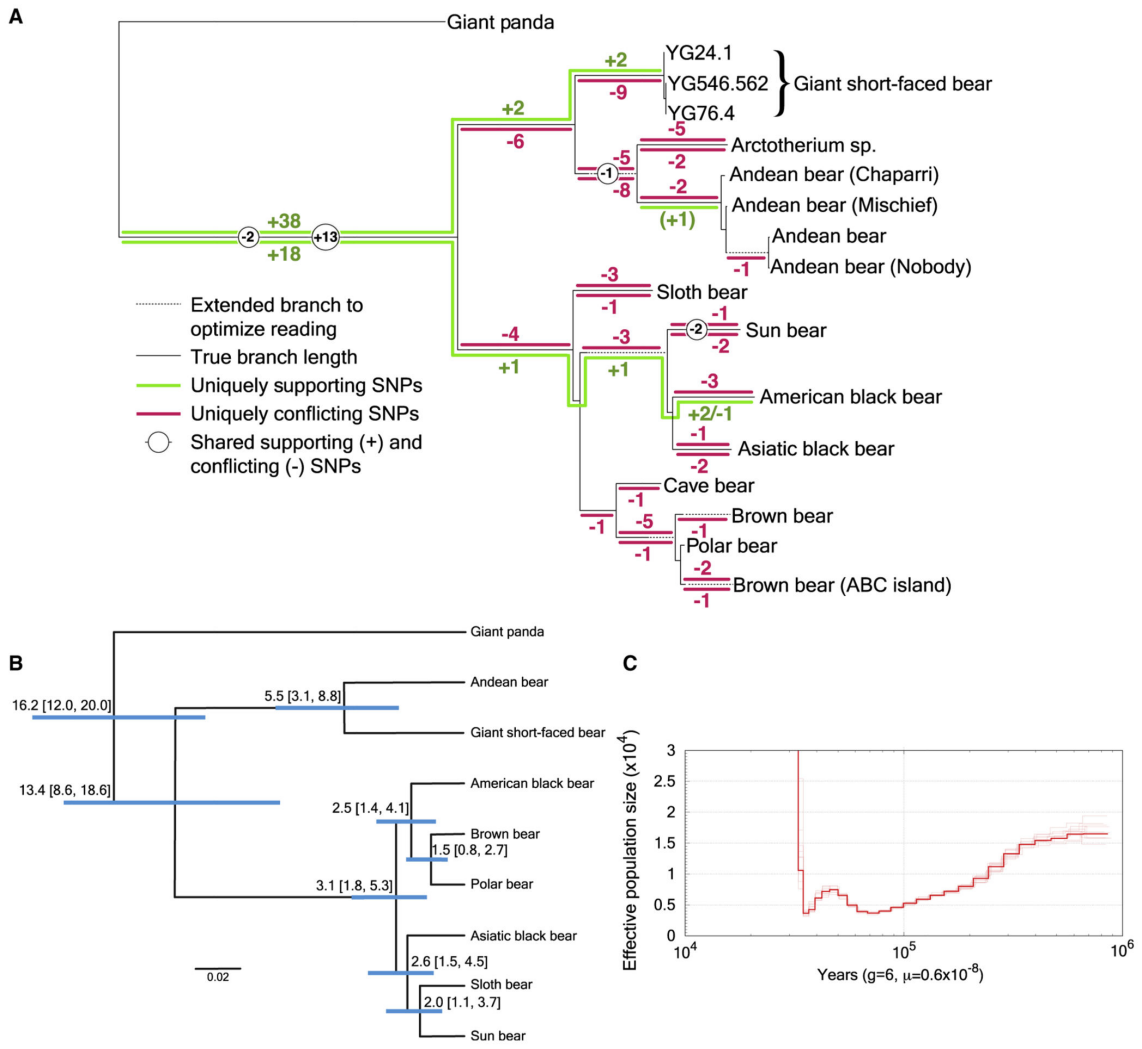
Giant short-faced bear genomic and population estimates (A) Biallelic transversion SNPs in UE1605, partitioned by read mapping (uniquely to the black bear mitochondrion, uniquely to Andean bear, or shared) and placed onto a mitochondrial Ursid tree. Lines above the black backbone lines of the tree indicate SNPs mapping uniquely to Andean bear; lines below the tree indicate mapping uniquely to black bear. The (+1) indicates a single supporting SNP in the black bear mapping leading to the Andean bear clade. (B) Phylogenetic tree and divergence times of the eight extant bear species and the extinct giant short-faced bear, as inferred from analysis of nuclear genomes. Branch lengths represent time before present (mya). The mean age of each node is shown, with 95% credibility intervals in parentheses and depicted as blue bars around each node. (C) PSMC plot for YG 546.562. See Figures S1 and S2 and Data S1.

Mitochondrial DNA analyses confirmed that the additional bear represented in UE1605 was a giant short-faced bear. We estimated a mitochondrial phylogeny using whole mitogenomes of the eight extant bears of the family Ursidae as well as three extinct bear lineages: cave bears (*U. spelaeus*) and the two extinct tremarctine bears, the North American giant short-faced bear, *Arctodus*, and the South American giant short-faced bear, *Arctotherium*, which we reassembled using the Andean bear as reference (Figure 4A; STAR Methods). To assign reads from UE1605 to this phylogeny, we implemented a competitive mapping approach in which we simultaneously mapped each read to both black bear and Andean bear mitochondrial genomes and partitioned them into read sets that (1) mapped uniquely to black bear, (2) mapped uniquely to *Tremarctos*, or (3) mapped to both. We then used *pathPhynder*^34^ to assign biallelic transversion SNPs onto the mitochondrial tree and to determine which SNPs in each read set either supported or conflicted with each branch of the phylogeny (Figure 4A). Apart from a single SNP from a read that mapped uniquely to the black bear and supports the Andean bear clade, which we assume is due to noise, this analysis supports two distinct paths on the mitochondrial phylogeny, one leading to the giant short-faced bear and the other to American black bear, confirming that the competitive mapping approach can separate two related species co-recovered from an eDNA sample. Indeed, we note that only 18 biallelic transversion SNPs assigned to branches mapped to both black bear and Andean bear mitochondrial genomes, despite their being species that diverged only ~13.4 million years ago (mya) (Figure 4B).

Although the mitochondrial data from UE1605 were too sparse to infer the evolutionary relationships between the Mexican and Yukon giant short-faced bear populations (only 197 reads mapped uniquely to the Andean bear mitochondrion), the nuclear data suggest that the two populations were genetically distinct. After filtering the UE1605 reads to obtain only those that mapped uniquely to the Andean bear reference genome, we investigated sites that were called as heterozygous transversions in the high coverage YG 564.562 sample, looking at pseudo-haploid calls made in the three low-coverage samples. The other Yukon samples, YG 24.1 and YG 76.4, showed a fraction of derived alleles of 31.1% and 37.9%, broadly consistent with the approximately one-third value expected if they came from a closely related population to the high-coverage sample, whereas the Mexican UE1605 sample had a derived allele fraction of 17.1%, indicating substantial divergence. We note that any bias introduced by the competitive mapping for UE1605 would have tended to favor derived alleles, suggesting this is, if anything, an overestimate.

Fossil remains from *Arctodus* are rare in North America despite its continuous existence on the continent through much of the Pleistocene. Geographically, its presence in Chiquihuite Cave also marks one of the most southerly findings of its distribution,^35,36^ which further underlines the value of eDNA records from rare taxa for our understanding of biogeography and the glacial refugia that existed during the LGM. Our nuclear genome-based phylogenetic analyses confirm that the North American giant short-faced bear is most closely related to extant Andean bear (Figure 4B), consistent with the findings of previous mitochondrial analyses,^37^ and show that these two taxa diverged ~5.5 mya. However, many details concerning the evolution and paleobiogeography of tribe Tremarctini are simply unknown, due to the limited macrofossil record.

We are unable to determine how many individuals contributed to our environmental genome. As we used a pseudo-haploid sequence for our analyses, which we obtained by selecting a random read at each position, our results concern the population from which these reads came. We note that this is true of all low-coverage genomes: because even a single individual is diploid, a pseudo-haploid genome from a fossil samples from two genomes from the population. All analyses used are robust to operating on a random sample of alleles from the population. We also note that if the sample arose from multiple individuals from different populations, for example due to gene flow and/or replacement, then the analyses would report results as for an admixed population. With sufficiently deep coverage it might in principle be possible to use linkage disequilibrium to distinguish a mixture of individuals from recent genetic admixture. In our case, neither the black bear nor *Arctodus* results suggested admixture.

## Conclusion

We present the first eDNA genomics study to show that it is possible to separate genomic-wide sequences from closely related species that are present in the same environmental samples, as long as reference data exist for the taxa in question. We further showcase how such an “environmental genome” can be used in population genomic and phylogenetic studies. This opens the possibility of analyzing DNA from environmental samples in a similar manner as is currently done for DNA from fossil remains. As fossils are valuable, DNA analyses are destructive, and most species and populations of the past are poorly represented in, or even absent from, fossil records, the analysis of ancient environmental genomes directly from eDNA will allow improved insights compared to what can be addressed by DNA from fossils alone.

## Star★Methods

Detailed methods are provided in the online version of this paper and include the following:

- KEY RESOURCES TABLE
- RESOURCE AVAILABILITY
  ○ Lead contact
  ○ Materials availability
  ○ Data and code availability
- EXPERIMENTAL MODEL AND SUBJECT DETAILS
  ○ YG 24.1
  ○ YG 76.4
  ○ YG 546.562
  ○ Chiquihuite Cave
- METHOD DETAILS
  ○ Environmental DNA laboratory methods
- QUANTIFICATION AND STATISTICAL ANALYSIS
  ○ Environmental DNA bioinformatic methods
  ○ Black bear analysis
  ○ Giant short-faced bear fossil analysis
  ○ Fossil giant short-faced bear data analysis
  ○ The Ursidae mitochondrial phylogeny
  ○ Giant short-faced bear eDNA analyses

## Star★Methods

## Key Resources Table

**Table:** 

REAGENT or RESOURCE	SOURCE	IDENTIFIER
Biological samples		
Osteological remain	This study	YG 76.4
Osteological remain	This study	YG 24.1
Osteological remain	This study	YG 546.562
Sediment sample	This study	UE1210_K3_SW_Mex_17_MWP1
Sediment sample	This study	UE1210_K3_SW_Mex_19_MWP15
Sediment sample	This study	UE1210_K3_SW_Mex_20_MWP16
Sediment sample	This study	UE1210_K3_SW_Mex_18_MWP2
Sediment sample	This study	UE1210_K3_SW_Mex_19_MWP3
Sediment sample	This study	UE1210_K3_SW_Mex_2_MWP2
Sediment sample	This study	UE1210_K3_SW_Mex_17_MWP13
Sediment sample	This study	UE1210_K3_SW_Mex-71_MWP19
Sediment sample	This study	UE1210_K3_SW_Mex-72_MWP20
Sediment sample	This study	UE1210_K3_SW_Mex-73_MWP21
Sediment sample	This study	UE1210_K3_SW_Mex-74_MWP22
Sediment sample	This study	UE1210_K3_SW_Mex-77_MWP26
Sediment sample	This study	UE1210_K3_SW_Mex-18_MWP13
Sediment sample	This study	UE1210_K3_SW_Mex-18_MWP13
Sediment sample	This study	UE1210_K3_SW_Mex-2_MWP2
Sediment sample	This study	UE1210_K3_SW_Mex-2_MWP2
Sediment sample	This study	UE1210_K3_SW_Mex-3_MWP3
Sediment sample	This study	UE1210_K3_SW_Mex-3_MWP3
Sediment sample	This study	UE1210_K3_SW_Mex-33_MWP42
Sediment sample	This study	UE1210_K3_SW_Mex-34_MWP43
Sediment sample	This study	UE1210_K3_SW_Mex-35_MWP44
Sediment sample	This study	UE1210_K3_SW_M105_ML105
Sediment sample	This study	UE1210_K3_SW_M106_ML106
Sediment sample	This study	UE1210_K3_SW_M107_ML107
Sediment sample	This study	UE1210_K3_SW_P_M108_ML108
Sediment sample	This study	UE1210_K3_SW_P_M109_ML109
Sediment sample	This study	UE1210_K3_SW_M123_ML125
Sediment sample	This study	UE1210_K3_SW_M124_ML126
Sediment sample	This study	UE1210_K3_SW_M125_ML127
Sediment sample	This study	UE1210_K3_SW_M126_ML128
Sediment sample	This study	UE1210_K3_SW_M127_ML129
Sediment sample	This study	UE1210_K3_SW_M129_ML131
Sediment sample	This study	UE1210_K3_SW_M130_ML132
Sediment sample	This study	UE1210_K3_SW_M131_ML133
Sediment sample	This study	UE1210_K3_SW_Mex-1_ML21
Sediment sample	This study	UE1210_K3_SW_Mex-1_ML22
Sediment sample	This study	UE1210_K3_SW_Mex-1_ML23
Sediment sample	This study	UE1210_K3_SW_Mex-2_ML24
Sediment sample	This study	UE1210_K3_SW_Mex-2_ML25
Sediment sample	This study	UE1210_K3_SW_Mex-2_ML26
Sediment sample	This study	UE1210_K3_SW_Mex-3_ML27
Sediment sample	This study	UE1210_K3_SW_Mex-3_ML28
Sediment sample	This study	UE1210_K3_SW_Mex-49_ML29
Sediment sample	This study	UE1210_K3_SW_Mex-72_ML31
Sediment sample	This study	UE1210_K3_SW_M85_ML85
Sediment sample	This study	UE1210_K3_SW_M86_ML86
Sediment sample	This study	UE1210_K3_SW_P_M88_ML88
Sediment sample	This study	UE1212_K3_SW_Mex_21_MWP5
Sediment sample	This study	UE1212_K3_SW_Mex_22_MWP6
Sediment sample	This study	UE1212_K3_SW_Mex_23_MWP7
Sediment sample	This study	UE1212_K3_SW_Mex_23_MWP19
Sediment sample	This study	UE1212_K3_SW_Mex_24_MWP20
Sediment sample	This study	UE1212_K3_SW_Mex_6_MWP5
Sediment sample	This study	UE1212_K3_SW_Mex_21_MWP17
Sediment sample	This study	UE1212_K3_SW_Mex_5_MWP1
Sediment sample	This study	UE1212_K3_SW_Mex_5_MWP3
Sediment sample	This study	UE1212_K3_SW_Mex-78_MWP27
Sediment sample	This study	UE1212_K3_SW_Mex-80_MWP29
Sediment sample	This study	UE1212_K3_SW_Mex-82_MWP31
Sediment sample	This study	UE1212_K3_SW_Mex-83_MWP32
Sediment sample	This study	UE1212_K3_SW_Mex-4_MWP34
Sediment sample	This study	UE1212_K3_SW_Mex-4_MWP34
Sediment sample	This study	UE1212_K3_SW_Mex-4_MWP35
Sediment sample	This study	UE1212_K3_SW_Mex-4_MWP36
Sediment sample	This study	UE1212_K3_SW_Mex-4_MWP36
Sediment sample	This study	UE1212_K3_SW_Mex-4_MWP37
Sediment sample	This study	UE1212_K3_SW_Mex-4_MWP37
Sediment sample	This study	UE1212_K3_SW_Mex-4_MWP38
Sediment sample	This study	UE1212_K3_SW_Mex-4_MWP38
Sediment sample	This study	UE1212_K3_SW_Mex-7_MWP39
Sediment sample	This study	UE1212_K3_SW_Mex-22_MWP14
Sediment sample	This study	UE1212_K3_SW_Mex-22_MWP14
Sediment sample	This study	UE1212_K3_SW_Mex-4_MWP4
Sediment sample	This study	UE1212_K3_SW_Mex-4_MWP4
Sediment sample	This study	UE1212_K3_SW_Mex-4_MWP45
Sediment sample	This study	UE1212_M4_SE_P_M110_ML110
Sediment sample	This study	UE1212_L3_SE_P_M14_ML18
Sediment sample	This study	UE1212_L3_SE_P_M15_ML19
Sediment sample	This study	UE1212_M4_SE_P_M24_ML20
Sediment sample	This study	UE1212_K3_SW_Mex-50_ML30
Sediment sample	This study	UE1212_M4_SE_P_M89_ML89
Sediment sample	This study	UE1212_L3_SE_P_M90_ML90
Sediment sample	This study	UE1605_B5_SW_P_Mex-94_MWP56
Sediment sample	This study	UE1605_B5_SW_P_Mex-94_MWP56
Sediment sample	This study	UE1605_B5_SW_P_M54_ML10
Sediment sample	This study	UE1605_B5_SW_P_M54_ML11
Sediment sample	This study	UE1605_B5_SW_P_M55_ML12
Sediment sample	This study	UE1605_B5_SW_P_M55_ML13
Sediment sample	This study	UE1605_B5_SW_P_M56_ML14
Sediment sample	This study	UE1605_B5_SW_P_M56_ML15
Sediment sample	This study	UE1605_B5_SW_P_M92_ML92
Sediment sample	^22^	UE1210_K3_SW_P_Mex-59_MWP59
Sediment sample	^22^	UE1210_K3_SW_Mex-18_MWP13
Sediment sample	^22^	UE1210_K3_SW_Mex-18_MWP13
Sediment sample	^22^	UE1210_K3_SW_Mex-2_MWP2
Sediment sample	^22^	UE1210_K3_SW_Mex_3_MWP3
Sediment sample	^22^	UE1210_K3_SW_Mex_3_MWP3
Sediment sample	^22^	UE1212_K3_SW_Mex-4_MWP45
Sediment sample	^22^	UE1212_K3_SW_P_Mex-58_MWP58
Sediment sample	^22^	UE1212_K3_SW_Mex_22_MWP14
Sediment sample	^22^	UE1212_K3_SW_Mex-24_MWP15
Sediment sample	^22^	UE1212_K3_SW_Mex_4_MWP2
Sediment sample	^22^	UE1212_K3_SW_Mex_5_MWP4
Sediment sample	^22^	UE1212_K3_SW_Mex_6_MWP5
Chemicals, peptides, and recombinant proteins		
N-Lauryl sarcosine sodium salt 100G	Sigma Aldrich	Cat# 8147150100
UltraPure Tris Hydrochloride 1L	Thermo Fisher Scientific	Cat# 15568025
UltraPure 0.5M EDTA, pH 8.0 100ML	Invitrogen	Cat# 15575-020
Ethanol absolute, Molecular Biology Grade, 250ML	VWR	Cat# 437433T
5M Sodium Chloride 1L	Sigma Aldrich	Cat#S5150
Water for Molecular Biology 500ML	Bioconcept	Cat# 3-07F04-I
2-Mercaptoethanol 100ML	Sigma Aldrich	Cat# M3148
UltraPure Dithiothreitol 5G	Thermo Fisher Scientific	Cat# 15508013
Proteinase K, recombinant, PCR Grade 25ML	Roche	Cat# 3115844001
EB Buffer	QIAGEN	Cat# 19086
PB Buffer	QIAGEN	Cat# 19066
PE Buffer	QIAGEN	Cat# 19065
Sodium acetate buffer solution 3M, pH 5.2 100ML	Sigma Aldrich	Cat# S7899
Phenol Red Solution 100ML	Sigma Aldrich	Cat# P0290
UltraPure Phenol:Chloroform:Isoamyl Alcohol 100ML	Thermo Fisher Scientific	Cat# 15593031
End Repair Module	NEBNext	Cat# E6050L
Quick ligation module	NEBNext	Cat# E6056L
Bst DNA polymerase	NEBNext	Cat# M0275L
dNTP set 100mM, 0.2ML	Geneon	Cat# 110-011
KAPA HiFi HotStart Uracil+ ReadyMix	Roche	Cat# KK2802
LightCycler 480 SYBR Green I Master	Roche	Cat# 4707516001
HighPrep PCR Clean-up System 50ML	MagBio	Cat# AC-60050
Critical commercial assays		
MinElute PCR Purification Kit	QIAGEN	Cat# 28006
Qubit dsDNA HS Assay Kit	Thermo Fisher Scientific	Cat# Q32854
Deposited data		
Sedimentary DNA sequence data	This study	ENA: PRJEB42692
Fossil Arctodus DNA sequence data	This study	ENA: PRJEB44161
Software and algorithms		
Admixtools	^26^	https://github.com/uqrmaie1/admixtools
bcftools	^38^	http://samtools.github.io/bcftools
Bowtie2	^39^	https://github.com/BenLangmead/bowtie2
Samtools	^40^	https://github.com/samtools/samtools
RaxML-ng	^41^	https://github.com/amkozlov/raxml-ng
Clustal omega	^42^	https://github.com/GSLBiotech/clustal-omega
PathPhynder	^34^	https://github.com/ruidlpm/pathPhynder
AdapterRemoval2.0	^43^	https://github.com/MikkelSchubert/adapterremoval
mapDamage2.0	^44^	https://github.com/ginolhac/mapDamage
EIGENSTRAT smartpca	^24^	https://github.com/DReichLab/EIG/tree/master/EIGENSTRAT
Phytools	^45^	https://github.com/liamrevell/phytools
PhyloMap	^25^	https://github.com/zhangjiajie/PhyloMap
PSMC	^46^	https://github.com/lh3/psmc
Plink	^47^	https://github.com/chrchang/plink-ng
Vcf to Eigenstrat format using a custom script	^48^	https://speciationgenomics.github.io/
PicardTools	^49^	https://github.com/broadinstitute/picard
BWA	^50^	https://github.com/lh3/bwa
MrBayes	^51^	https://github.com/NBISweden/MrBayes
OxCal	^52^	https://c14.arch.ox.ac.uk/oxcal.html
Partitionfinder	^53^	https://github.com/brettc/partitionfinder
GATK	^54^	https://github.com/broadinstitute/gatk
EMBOSS Cons	^55^	https://www.ebi.ac.uk/Tools/msa/emboss_cons/
pheatmap	^56^	https://github.com/raivokolde/pheatmap
bedtools	^57^	https://github.com/arq5x/bedtools2
SNP-sites	^58^	https://github.com/sanger-pathogens/snp-sites
MCMCTree	^59^	http://abacus.gene.ucl.ac.uk/software/paml.html
mia	^60^	https://github.com/mpieva/mapping-iterative-assembler

### Resource Availability

#### Lead contact

Further information and requests for resources and reagents should be directed to and will be fulfilled by the lead contact, Eske Willerslev, (ew482@cam.ac.uk).

#### Materials availability

Sequence data has been deposited at the European Nucleotide Archive under ENA accession number PRJEB42692 and PRJEB44161.

## Experimental Model and Subject Details

We studied three fossil individuals from an extinct giant-short faced bear population in the Yukon Territory. These were found at three localities across Yukon and are now curated by The Yukon Beringia Interpretive Centre.

### YG 24.1

YG 24.1 a complete cranium of a giant short-faced bear (*A. simus*) that was collected from Pleistocene age permafrost sediments exposed at a placer gold mine along Ophir Creek, Yukon Territory, Canada. All measurements on the cranium demonstrate this is a very small individual compared to other specimens of this species which have been described from the region. All sutures appear to be fused and all adult teeth are present and fully erupted, demonstrating this individual was an adult. As a high degree of sexual dimorphism has been demonstrated for *A. simus*, it is likely this small cranium represents an adult female.

### YG 76.4

YG 76.4, a complete radius bone from a giant short-faced bear (*A. simus*) that was collected from Pleistocene age permafrost sed-iments exposed at a placer gold mine along Hester Creek, Yukon Territory, Canada. The very large size of this radius precludes it from being any other large Pleistocene carnivore known from the region. The bone exhibits a high degree of bone exostosis on major muscle attachments, suggesting this represents an older adult male. This radius (YG 76.4) articulates with specimen YG 129.1, a complete right ulna, which was collected at the same locality.

### YG 546.562

YG 546.562, a small fragment of a right femur diaphysis collected from Pleistocene age permafrost sediments exposed at a placer gold mine along Canyon Creek, Yukon Territory, Canada. The thick cortical bone wall and curvature of the diaphysis clearly compare well with those of a giant short-faced bear (*A. simus*). See Table S3 for measurements and radiocarbon ages.

### Chiquihuite Cave

All sediment deposits and layers from UE1210 and UE1212 along with a detailed description of the cave have already been described elsewhere in Ardelean et al.^22^ UE1605 is a layer found closer toward the entrance of the cave. To determine the age of UE1605 we collected three charcoal samples from within the layer, which were AMS radiocarbon dated at the Oxford Radiocarbon Accelerator Unit (ORAU). The three samples yielded radiocarbon ages of 11,419 ± 34 (OxA-38748), 11,942 ± 33 (OxA-38746), and 12,901 ± 75 (OxA-X-3036-30) which corresponds to a calibrated age of between 15.0-13.0 cal kyr BP for UE1605. The age of layers UE1210 and UE1212 was determined by radiocarbon dating.^22^

We sampled all layers *in situ* using ancient DNA precautions, wearing face masks, hairnets, a full-body suit, boot covers, and nitrile gloves, and transferring the sediment to clean either sterile 50-mL spin tubes or 0.5-L plastic containers using sterile disposable scalpels or cleaned metal spoons. Samples were hereafter sent to Copenhagen and stored at −20 °C until further subsampling and extraction.

## Method Details

### Environmental DNA laboratory methods

All DNA extractions, library- and indexing PCR reactions were undertaken in ancient DNA dedicated facilities at the Lundbeck Foundation Centre for GeoGenetics, Copenhagen Denmark. Between 4-7 g of sediments were extracted for DNA using a Tris-HCl and 230 μg proteinase-K-based buffer (“Sergey Bulat buffer”), and purified using an organic extraction method.^4^ First, all samples were vigorously shaken to lyse and release DNA from tissue and minerals, using a FastPrep at 4.5 m/s for 40 s and thereafter incubated with gentle rotation overnight at 37°C. All samples were then spun at 4.000 g for 15 min and the supernatant was transferred to a sterile 15 mL spin filter. Ten mL of UltraPure Phenol:Chloroform:Isoamyl-alcohol (25:24:1) were added to the retained volume, and incubated at room temperature for 10 min while gently rotating. All samples were then centrifuged at 4,000 g for 5 min and the supernatant transferred to fresh 10 kDa Amicon Ultra-15 filters. The samples were then spun at 4,000 g to a 200 μL volume and washed twice with 1.0 mL QIAGEN EB buffer and spun to a 200 μL volume. The final retentate was then transferred to a sterile low-bind Eppendorf tube and stored at −20°C until further downstream processing. All extracts were hereafter converted into a total of 72 dual-indexed Illumina libraries using standard protocol^61^ and sequenced on an Illumina HiSeq 4000 80bp single-read or NovaSeq 6000 platform 100bp paired-end.

## Quantification and Statistical Analysis

### Environmental DNA bioinformatic methods

Upon sequencing all data were demultiplexed, trimmed, overlapping read pairs collapsed using AdapterRemoval v2^43^ and merged by layer together with the 15 Illumina libraries recently published in Ardelean et al.^22^ The total of 55,845,081,142 reads from all 87 libraries were hereafter parsed and mapped for further downstream analysis. Throughout we used bowtie2^39^ for read mapping, with parameters to increase sensitivity but restricting to end-to-end alignments (-D 20 -R 3 -N 1 -L 20 -i S,1,0.50–end-to-end–no-unal) and parsing only aligned reads.

We first performed a competitive mapping against the RefSeq mitochondrial genome database (Database: RefSeq version 92 mitochondrial genomes) to taxonomically classify mammalian DNA, which confirmed the previous finding of American black bear (*U. americanus*) in layer UE1210 and UE1212, as well as the presence of both the giant short-faced bear (*Arctodus simus*) and American black bear in layer UE1605.

We next mapped all trimmed and collapsed reads separately against all available mitochondrial genomes of bears: American black bear (*Ursus americanus* (Genbank: NC_003426.1, NC_003426.1)), brown bear *(Ursus arctos*, (Genbank: NC_003427.1)), Andean bear (*Tremarctos ornatus*, NC_009969.1), polar bear (Ursus maritimus, NC_003428.1, Asian black bear (*Ursus thibetanus*, (Genbank: NC_008753.1, NC_009331.1, NC_009971.1, NC_011117.1, NC_011118.1)), giant panda (*Ailuropoda melanoleuca*, (NC_009492.1)), sun bear (*Helarctos malayanus*, (NC_009968.1)), sloth bear (*Melursus ursinus*, (Genbank: NC_009970.1)), cave bear (*Ursus spelaeus*, (Genbank: NC_011112.1)), short-faced bear (*Arctotherium sp*., (Genbank: KU886001.1)), and the giant short-faced bear (*Arctodus simus*, (NC_011116.1)). We then mapped all reads against the full reference genomes of the Andean bear (Tremarctos ornatus) - the closest relative to the giant short-faced bear^40^ (Genbank: WMLG00000000), and the American black bear (U. americanus, (Genbank: GCA_003344425.1)), as well as to the polar bear (U. maritimus, (Genbank: GCA_000687225.1)) and giant panda (A. melanoleuca, (Genbank: GCF_002007445.1)) genomes for further authentication (Figure S1B). All alignments were hereafter filtered for quality score of ≥25 and parsed for further downstream phylogenetic placement and population genetic analysis. This resulted in between 1-1.6 million reads aligning to American black bear with a coverage of 0.025x, 0.019x and 0.033x for UE1210, UE1212 and UE1605, respectively, as well as a coverage for giant short-faced bear of 0.041x for UE1605.

### Black bear analysis

To contextualize the ancient black bear genome-wide data, we realigned the original fastq files from the RAD-seq dataset of 83 modern black bears from across the United States^23^ against the black bear reference genome (Genbank: LZNR01000000^62^) using bwa aln with default parameters (Li and Durbin, 2010). We called a vcf using bcftools with default parameters^38^ and filtered for a read mapping quality of > 20 and AN > 150, that is, those sites which were covered by at least 90% or 75 of the 83 individuals, using samtools.^40^ The latter was done to ensure we used variants for which the majority of the samples had genetic information and resulted in 101,961 SNPs to be parsed for phylogenetic analysis.

We next used Plink^47^ to create a distance matrix of only the modern samples, then constructed a neighbor-joining tree with all modern samples in R using the *phytools* package.^45^ Genomic coordinates were then called using Plink to generate a .bed file of coordinates containing biallelic SNPs according to the vcf of the modern samples. On these coordinates, a pileup was created on the three ancient samples and converted to Plink format using a custom Python script. This resulted in 2646 pseudo haploid SNPs for UE1210, 1927 for UE1212, and 2954 for UE1605. We next merged the modern and ancient Plink files, and used EIGENSTRAT’s *smartpca*^24^ with shrinkmode and lsqproject options to project the ancient samples onto the modern variation. PC1 accounted for 5.13% of the variation and PC2 accounted for 2.94%. We plotted the figure rotated to approximately correspond with the geographical structure of the populations (Figure 1B).

To measure the relative genetic distance of each ancient sample to each of the modern individuals, we merged all Plink files and created a pairwise genetic Hamming distance matrix on biallelic SNPs with the flat-missing modifier. For the missing values in the ancient samples, Plink rescales the distances to be on the same scale as the rest of the matrix. We then mapped the scaled distances of each ancient sample to each modern sample onto a color scale, and plotted the colors on a *phylomap*^25^ plot to visualize the distance of each ancient sample to each modern sample. The phylogenetic tree shown in this plot is the neighbor-joining tree produced by a distance matrix of only the modern samples using Plink. This is shown in Figure 1C for UE1210, with additional figures in the supplement for other two samples (Figure S3B).

To calculate *f4* statistics and create an admixture graph, we first needed an outgroup on the same coordinates as the black bear reference genome. We mapped two polar bear short read genomes (Genbank: SAMN01057659 and SAMN01057636)^63^ onto the black bear reference genome^62^ using bwa mem^50^ with default parameters, and filtered for read quality > 30. We compiled the two polar, the 83 modern, and the three ancient black bears into a single vcf file using bcftools.^64^ We labeled samples as belonging to one of the following populations: Polar, Mexican, East, Southwest, Kenai (Alaska West), and SEAK (Southeast Alaska), where Mexican refers to the ancient samples and the other labels and groupings were decided using both phylogenetic and geographic factors of the modern population, and previous literature.^23^ We removed the Northwest population since that population was concluded to be admixed in previous literature^23^ and therefore may unnecessarily complicate the current analysis. We also removed three SEAK samples from the southern Alexander Archipelago that clustered separately from other SEAK samples in the PCA and phylogenetic analysis (Figures 1B and 1C) as well as in previous coancestry heatmaps.^23^ We converted this vcf to Eigenstrat format using a custom script from Ravinet and Meier,^48^ and used the *admixtools* package^26^ in R to compute f4 statistics. We filtered the results to only those which included the polar bear outgroup and these are shown in Table S2.

On the same dataset, we then used the qpGraph function in the *admixtools* package in R to determine an admixture graph. We used the maxmiss = 1 and afprod = TRUE options, with 500 SNP blocks for the jackknife, and default options otherwise. We first used automatic graph optimization, allowing for one admixture edge, to determine a graph using the East, Southwest, Kenai (West Alaska), SEAK (Southeast Alaska), Mexican and polar populations. Since this graph fit poorly and had excess f4 residuals with z-scores over 6, we added another admixture edge at all possible positions, resulting in seven highest-scoring graphs with similar topologies that fit the data well, each with a maximum excess f4 residual of |Z| = 2.182. Each of these graphs agreed on some basic structural characteristics, including a deep split between Mexican/East/Kenai and Southwest/SEAK, with Mexican basal on the Mex/East/Kenai side, and with both Southwest and SEAK admixed. Furthermore, in each graph the SEAK population took most of its admixture from the Mexican/East/Kenai clade, from a population most closely related to Kenai. We show the highest-scoring of these graphs, with a score of 4.922, in Figure 1D, and the remaining six in Figure S4.

### Giant short-faced bear fossil analysis

To aid phylogenetic placement and separation of the reads from both bear species, we analyzed three *Arctodus simus* fossil bones recovered from permafrost sediments in Yukon Territory, Canada (Figure 3; Data S1). These included a petrous bone from a complete cranium from Ophir Creek near Dawson Creek (YG 24.1), a complete radius from Hester Creek (YG 76.4), and a fragment of a right femur from Canyon Creek (YG 546.562). We subsampled for DNA and radiocarbon dating using a handheld rotating cutting tool and individually submitted samples for radiocarbon dating to the Keck Carbon Cycle AMS facility at the University of California, Irvine (UCIAMS). The three obtained ages were hereafter calibrated using OxCal (version 4.3)^52^ with the northern hemisphere atmospheric radiocarbon curve,^65^ and all yielded Late Pleistocene ages, YG 24.1 (49.8 cal kyr BP, UCIAMS 186674), YG 76.4 (30.8 cal kyr BP, CAMS-166313), YG 546.562 (31.8 cal kyr BP, UCIAMS 186671).

The subsamples for DNA extraction were ground to powder using a Mixer Mill MM 400 (Retsch), with the exception of YG 24.1 which was received at UC Santa Cruz as powder. We performed DNA extraction and library preparation in the ancient DNA dedicated facilities at UC Santa Cruz, following ancient DNA precautions.^38^ To increase endogenous content, powder from YG 24.1 and YG 76.4 was pretreated with a bleach solution following an established protocol.^66^ Between 50-120 mg of bone powder from each sample was incubated rotating overnight (~18-24 h) at 37°C in 1mL of lysis buffer (0.45M EDTA, 0.25mg/mL Proteinase K), after which the DNA was isolated using the silica column based Dabney method^67^ and eluted in 50 μL of buffer EBT (10mM Tris, 0.05% Tween-20).

We converted the extracted DNA from YG 24.1 and YG 76.4 into one double-stranded DNA (dsDNA) library each using established protocols,^61^ while starting with a template volume of 20 μL and following the modifications in the Pennsylvania State University library preparation protocol.^68^ Indexing PCR was performed for 25 cycles in 50 μL reactions using AmpliTaq Gold polymerase in buffer II. All dsDNA libraries were hereafter pooled and sequenced on an Illumina HiSeq 2500 (2x 50bp) at the UC San Francisco Center for Advanced Technology.

For specimen YG 546.562, we prepared single-stranded DNA (ssDNA) libraries using an ancient DNA optimized version of the method outlined in Kapp et al.^69^ Quantitative PCR was used to determine the number of cycles for each indexing PCR. All ssDNA libraries were indexed and amplified in 100 μL reactions containing 48 μL pre-amplified library, 50 μL Ampli-Taq Gold 360 Master Mix, 1 μM i7 indexing primer, and 1 μM i5 indexing primer. Post-amplified libraries were next purified using a 1:1.2 library:SPRI beads ratio and DNA concentration quantified on a Qubit 4 (Invitrogen) using the Qubit 1X dsDNA HS assay kit. Lastly, all post-amplified libraries were visualized on a Fragment Analyzer (Agilent) using a HS NGS Fragment Kit (1-6000bp) Assay (Agilent), pooled, and sequenced on an Illumina NovaSeq S4 (2x 100bp) at the UC San Francisco Center for Advanced Technology.

### Fossil giant short-faced bear data analysis

Raw reads were trimmed for adapters and read pairs merged using SeqPrep2, following default parameters with a quality score cut-off of 15 (-q 15) and with an overlap of 20bp for YG 24.1 and YG 76.4, and YG 546.562 with a 15bp overlap. In addition, we used Trimmomatic^70^ to remove residual adapters from the merged reads, setting a seed mismatch of 2 and a simple clip threshold to 4 for short adaptor sequences. Further, all ends were trimmed for base qualities using minimum quality of 2 and 5 for leading and trailing end of the reads, respectively. Lastly, we used a sliding window to quality trim bases of the size of 4 with less than a quality of 15 and parsed only reads ≥35bp. We next mapped all reads against the *Tremarctos ornatus* genome (Genbank: WMLG00000000)^71^ using bwa aln^50^ (-l 1024 -n 0.01 -o 2), parsing reads with a mapping quality ≥30 and removing PCR duplicates using samtools rmdup.^40^ This resulted in a total of 43,107,072, 50,492,295 and 758,541,872 reads aligning to the *Tremarctos ornatus* genome with a coverage of 1.82x, 1.66x and 26.01x, for YG 24.1, YG 76.4, and YG 546.562, respectively. Finally, we realigned reads around insertions and deletions using GATK Realigner Target Creator and Indel Realignment tools.^54^ We next parsed the alignment using MapDamage2.0^44^ to assess the frequency of 5′ and 3′ substitutions and found that all three samples showed elevated deamination at the first positions (> 0.13), which is characteristic of ancient DNA (Figures S2A–S2C).

We used the pairwise sequentially Markovian coalescent (PSMC) model^46^ to estimate the historical effective population size of YG 546.562 (Figure 4C). The input was a realigned alignment file mapped to the Andean bear genome, where scaffolds less than 1Mb in length were removed. We used sites between one third and twice the average coverage. We used a generation time of 6 years and a mutation rate of 0.6e-8 per bp per generation, based on previous estimates of the ursid mutation rate.^72^ To account for the age of our ancient sample, we rescaled the x axis, by adding the calibrated age (29,242 cal. year BP) to the scaled time points, thus pushing back the start of the PSMC model to 29,242 ya. We also performed ten bootstrap replicates, scaling each by the sample age per the software instructions.^46^

Our high coverage short-faced bear genome also allowed us to estimate a timeline for the divergence between tremarctine and ursine bears (Figure 4B). From the high-coverage short-faced bear genome and published genomes of the eight extant bears in the ursid family, we extracted a set of 13,713 single copy orthologous coding sequences, based on annotations of the giant panda and polar bear genomes.^73,74^ We generated fasta sequences from each of the eight extant bear species and the giant short-faced bear for only the four-fold degenerate codon positions, resulting in a total of 3,415,480 bases per bear. We estimate divergence times among the bear species using an approximate likelihood calculation with MCMCTree^59^ under an independent clock model with one fossil calibration and one tip date for the giant short-faced bear (Figure 4B).

### The Ursidae mitochondrial phylogeny

We next sought to place the fossil bear DNA in a maximum likelihood (ML) and Bayesian mitochondrial phylogeny of all eight extant and three extinct bear species by first parsing all reads from our samples aligned to the mitochondrial genomes, with a reduced minimum length of 25 bp (-L 25) to increase coverage using SeqPrep2. Initially, we assembled full mitochondrial genomes of all three short-faced bear fossils, using the mitochondrial assembler mia,^60^ and the publicly available *Arctodus simus* mitochondrial genome as the reference (Genbank: NC011116.1),^37^ with an ancient DNA substitution matrix. This resulted in assembly coverage of 66.12x, 19.22x and 44.88x for YG 24.1 and YG 76.4, and YG 546.562, respectively. We further downloaded a publicly available dataset of ~1.5 million reads from an mitochondrial enriched genomic library of *Arctotherium sp*. from Chile^75^ and reassembled the mitochondrial genome using the Andean bear as reference (Genbank: FM177764.1) following the method outlined above. This resulted in a mitochondrial coverage for *Arctotherium* of 126x a consensus genome for all assemblies was created using a threshold of 66% and 3X coverage per site. Any site not meeting these criteria was changed to an ‘N’.

For the remaining bears, sun bear, sloth bear, cave bear, Asiatic black bear, giant panda, brown bear, polar bear, Andean bear, ABC island brown bear, and American black bear we downloaded publicly available mitochondrial sequences for the construction of a mitochondrial phylogeny (Genbank: FM177765.1, FM177763.1, FM177760.1, FM177759.1, EF212882.1, EU497665.1, GU573490.1, FM177764.1, JX196368.1, AF303109.1, respectively).

We used clustal omega^42^ to align the bear mitochondrial genomes and partitioned the alignment into six datasets: free sites, control region, rRNA, tRNA, 1st and 2nd coding positions and 3rd coding positions based on annotations of the Asiatic black bear (Gen-bank: FM177759.1), polar bear (Genbank: GU573490.1), and Andean bear (Genbank: FM177764.1) from Geneious.^76^ We hereafter ran PartitionFinder^53^ to determine the partitions and best substitution model, with branch lengths unlinked using the Bayesian Information Criterion. PartitionFinder separated the data into three mitochondrial partitions; 1) control region (Hasegawa-Kishino-Yano + gamma), 2) free sites, rRNA, tRNA, 1st and 2nd coding positions (general time reversible + gamma + invariable sites), and 3) 3rd codon positions (general time reversible + gamma + invariable sites).

We then performed a ML and Bayesian phylogenetic analyses using the partitioning specified above. RAxML^77^ was used to produce a ML phylogeny, running one hundred bootstrap replicates. We created a phylogeny using a Bayesian approach using MrBayes^51^ with the same partitioning as above. We ran four chains (one hot, three cold) for 10 million generations, with trees and model parameters sampled every 1,000 generations, with the first 25% discarded as burn-in.

### Giant short-faced bear eDNA analyses

Next we sought to contextualise the giant short-faced bear eDNA sample UE1605 by placing it phylogenetically in the wider ursid tree. From our multiple sequence alignment of 14 ursid mitochondrial genomes, which included the three *Arctodus* fossil mitochondria, we created a vcf using SNP-sites with default parameters,^58^ and filtered out sites which contained non-ACTG bases in the reference or were not biallelic, which left 5071 sites. We also called a consensus sequence of length 16981 sites on the Ursid mitochondrion multiple sequence alignment using EMBOSS cons with default parameters.^55^

To place our ancient environmental sample UE1605 phylogenetically, we used a software called pathPhynder.^34^ Since our eDNA samples contain both black bear and giant short-faced bear DNA, we used Picard’s^49^ FilterSamReads function to partition the .bam files into three sets: reads that mapped uniquely to the Andean bear reference mitochondrion (Genbank: NC_011116.1^37^), reads that mapped uniquely to the black bear reference mitochondrion (Genbank: NC_003426.1^39^), and reads that mapped to both. We then used bedtools bamtofastq^57^ to convert each of these read sets back to fastq format, and then bwa aln -l 1024 -n 0.02 (ancient DNA parameters^50^) to re-map these reads to the consensus ursid sequence, because we needed our ancient sample to be on the same coordinate system as the reference multiple sequence alignment. We then gave as input to pathPhynder the ursid phylogenetic tree, the filtered ursid vcf file, the ursid mitochondrion consensus sequence, and our UE1605 read sets mapped to the consensus. We used the best-path mode and the transversion only filter and otherwise default parameters. For each read set, we ran a custom Perl script on the pathPhynder output, and thus were able to determine which biallelic transversion SNPs in our UE1605 sample mapped to Andean bear uniquely, black bear uniquely, or both, and which of each of these were in support or conflict on each branch of the phylogeny (Figure 4A).

We next wanted to compare UE1605 and the three fossil giant short-faced bears on the nuclear genome. First, we called a vcf of biallelic transversion SNPs on the high coverage sample YG 546.562 using bcftools,^38^ and looked specifically at heterozygous sites filtered using samtools call quality > 20, mapping quality > 25, and depth between 15 and 40. On these high-quality heterozygous sites, we counted pseudohaploid calls in the three low coverage samples YG76.4, YG 24.1 and UE1605, obtained by selecting the allele matched when only one read overlapped the site, which avoids issues of potential amplification bias. We found an alternate allele fraction of 37.89% for YG 76.4, 31.12% for YG 24.1, and 17.10% for UE1605, where YG 24.1 is ~20ka older, indicating that the Mexican giant short-faced bear is substantially more than 20ka diverged from the YG 546.562 and YG 76.4 samples.

## Supplementary Material

Data S1

Figure S1, Figure S2, Figure S2, Figure S4

Table S1

Table S2

## Data Availability

The accession number for the genomic data reported in this paper is ENA: PRJEB42692 and PRJEB44161. All code used in this study and other previously published genomic data is available at the sources referenced in key resource table.

## Highlights

- We recovered genomic data from ancient bears directly from cave sediments
- Ancient Mexican black bears are ancestrally related to Eastern American black bears
- The Mexican and Yukon populations of extinct giant short-faced bears were distinct
- Our results bring ancient eDNA into the era of population genomics

## In brief

To date, ancient eDNA has been limited to simple taxonomic profiling of biological communities rather than population or phylogenetic studies. Using genome-wide analysis of American black bear and giant short-faced bear DNA extracted from cave sediments, Pedersen et al. show that population genomics is now possible using ancient eDNA.

## References

[1] Willerslev E, Hansen AJ, Binladen J, Brand TB, Gilbert MTP, Shapiro B, Bunce M, Wiuf C, Gilichinsky DA, Cooper A (2003). Diverse plant and animal genetic records from Holocene and Pleistocene sediments. Science.

[2] Willerslev E, Hansen AJ, Poinar HN (2004). Isolation of nucleic acids and cultures from fossil ice and permafrost. Trends Ecol Evol.

[3] Willerslev E, Cappellini E, Boomsma W, Nielsen R, Hebsgaard MB, Brand TB, Hofreiter M, Bunce M, Poinar HN, Dahl-Jensen D (2007). Ancient biomolecules from deep ice cores reveal a forested southern Greenland. Science.

[4] Pedersen MW, Ruter A, Schweger C, Friebe H, Staff RA, Kjeldsen KK, Mendoza MLZ, Beaudoin AB, Zutter C, Larsen NK (2016). Postglacial viability and colonization in North America’s ice-free corridor. Nature.

[5] Seersholm FV, Pedersen MW, Søe MJ, Shokry H, Mak SST, Ruter A, Raghavan M, Fitzhugh W, Kjær KH, Willerslev E (2016). DNA evidence of bowhead whale exploitation by Greenlandic Paleo-Inuit 4,000 years ago. Nat Commun.

[6] Slon V, Hopfe C, Weiß CL, Mafessoni F, de la Rasilla M, Lalueza-Fox C, Rosas A, Soressi M, Knul MV, Miller R (2017). Neandertal and Denisovan DNA from Pleistocene sediments. Science.

[7] Ahmed E, Parducci L, Unneberg P, Ågren R (2018). Archaeal community changes in Lateglacial lake sediments: evidence from ancient DNA. Quat Sci Rev.

[8] Murchie TJ, Kuch M, Duggan AT, Ledger ML, Roche K, Klunk J, Karpinski E, Hackenberger D, Sadoway T, MacPhee R (2021). Optimizing extraction and targeted capture of ancient environmental DNA for reconstructing past environments using the PalaeoChip Arctic-1.0 bait-set. Quat Res.

[9] Freeland JR (2017). The importance of molecular markers and primer design when characterizing biodiversity from environmental DNA. Genome.

[10] Lammers Y, Heintzman PD, Alsos IG (2021). Environmental palaeogenomic reconstruction of an Ice Age algal population. Commun Biol.

[11] Schulte L, Bernhardt N, Stoof-Leichsenring K, Zimmermann HH, Pestryakova LA, Epp LS, Herzschuh U (2021). Hybridization capture of larch (Larix Mill) chloroplast genomes from sedimentary ancient DNA reveals past changes of Siberian forest. Mol Ecol Resour.

[12] Shapiro B, Hofreiter M (2014). A paleogenomic perspective on evolution and gene function: new insights from ancient DNA. Science.

[13] Nielsen R, Akey JM, Jakobsson M, Pritchard JK, Tishkoff S, Willerslev E (2017). Tracing the peopling of the world through genomics. Nature.

[14] Shepherd LD (2017). A non-destructive DNA sampling technique for herbarium specimens. PLoS ONE.

[15] Thomsen PF, Elias S, Gilbert MTP, Haile J, Munch K, Kuzmina S, Froese DG, Sher A, Holdaway RN, Willerslev E (2009). Non-destructive sampling of ancient insect DNA. PLoS ONE.

[16] Sponheimer M, Ryder CM, Fewlass H, Smith EK, Pestle WJ, Talamo S (2019). Saving old bones: a non-destructive method for bone collagen prescreening. Sci Rep.

[17] Taberlet P, Coissac E, Hajibabaei M, Rieseberg LH (2012). Environmental DNA. Mol Ecol.

[18] Thomsen PF, Willerslev E (2015). Environmental DNA – an emerging tool in conservation for monitoring past and present biodiversity. Biol Conserv.

[19] Pedersen MW, Overballe-Petersen S, Ermini L, Sarkissian CD, Haile J, Hellstrom M, Spens J, Thomsen PF, Bohmann K, Cappellini E (2015). Ancient and modern environmental DNA. Philos Trans R Soc Lond B Biol Sci.

[20] Stahlschmidt MC, Collin TC, Fernandes DM, Bar-Oz G, Belfer-Cohen A, Gao Z, Jakeli N, Matskevich Z, Meshveliani T, Pritchard JK (2019). Ancient mammalian and plant DNA from Late Quaternary stalagmite layers at Solkota Cave, Georgia. Sci Rep.

[21] Graham RW, Belmecheri S, Choy K, Culleton BJ, Davies LJ, Froese D, Heintzman PD, Hritz C, Kapp JD, Newsom LA (2016). Timing and causes of mid-Holocene mammoth extinction on St. Paul Island, Alaska Proc Natl Acad Sci USA.

[22] Ardelean CF, Becerra-Valdivia L, Pedersen MW, Schwenninger J-L, Oviatt CG, Macías-Quintero JI, Arroyo-Cabrales J, Sikora M, Ocampo-Díaz YZE, Rubio-Cisneros II (2020). Evidence of human occupation in Mexico around the Last Glacial Maximum. Nature.

[23] Puckett EE, Etter PD, Johnson EA, Eggert LS (2015). Phylogeographic analyses of American black bears (Ursus americanus) suggest four glacial refugia and complex patterns of postglacial admixture. Mol Biol Evol.

[24] Patterson N, Price AL, Reich D (2006). Population structure and eigenanalysis. PLoS Genet.

[25] Zhang J, Mamlouk AM, Martinetz T, Chang S, Wang J, Hilgenfeld R (2011). PhyloMap: an algorithm for visualizing relationships of large sequence data sets and its application to the influenza A virus genome. BMC Bioinformatics.

[26] Maier R (2020). Admixtools.

[27] Wang X, Rybczynski N, Harington CR, White SC, Tedford RH (2017). A basal ursine bear (Protarctos abstrusus) from the Pliocene High Arctic reveals Eurasian affinities and a diet rich in fermentable sugars. Sci Rep.

[28] Rybczynski N, Gosse JC, Harington CR, Wogelius RA, Hidy AJ, Buckley M (2013). Mid-Pliocene warm-period deposits in the High Arctic yield insight into camel evolution. Nat Commun.

[29] Pelletier A, Obbard ME, Mills K, Howe EJ, Burrows FG, White BN, Kyle CJ (2012). Delineating genetic groupings in continuously distributed species across largely homogeneous landscapes: a study of American black bears (Ursus americanus) in Ontario, Canada. Can J Zool.

[30] Onorato DP, Hellgren EC, van den Bussche RA, Doan-Crider DL (2004). Phylogeographic patterns within a metapopulation of black bears (Ursus americanus) in the American Southwest. J Mammal.

[31] Onorato DP, Hellgren EC, Van Den Bussche RA, Doan-Crider DL, Skiles JR (2007). Genetic structure of American black bears in the desert southwest of North America: conservation implications for recolonization. Conserv Genet.

[32] Varas-Nelson AC (2010). Conservation genetics of black bears in Arizona and northern Mexico (University of Arizona).

[33] Sorkin B (2006). Ecomorphology of the giant short-faced bears Agriotherium and Arctodus. Hist Biol.

[34] Martiniano R, De Sanctis B, Hallast P, Durbin R (2020). Placing ancient DNA sequences into reference phylogenies. bioRxiv.

[35] Luna-Aranguré C, Soberón J, Vázquez-Domínguez E (2020). A tale of four bears: environmental signal on the phylogeographical patterns within the extant Ursus species. J Biogeogr.

[36] Arroyo-Cabrales J, Johnson E, Graham RE, Pérez-Crespo VA (2016). North American ursid (mammalia: ursidae) defaunation from Pleistocene to recent. Cranium.

[37] Krause J, Unger T, Noçon A, Malaspinas A-S, Kolokotronis S-O, Stiller M, Soibelzon L, Spriggs H, Dear PH, Briggs AW (2008). Mitochondrial genomes reveal an explosive radiation of extinct and extant bears near the Miocene-Pliocene boundary. BMC Evol Biol.

[38] Cooper A, Poinar HN (2000). Ancient DNA: do it right or not at all. Science.

[39] Langmead B, Salzberg SL (2012). Fast gapped-read alignment with Bowtie 2. Nat Methods.

[40] Li H, Handsaker B, Wysoker A, Fennell T, Ruan J, Homer N, Marth G, Abecasis G, Durbin R, 1000 Genome Project Data Processing Subgroup (2009). The Sequence Alignment/Map format and SAMtools. Bioinformatics.

[41] Kozlov AM, Darriba D, Flouri T, Morel B, Stamatakis A (2019). RAxML-NG: a fast, scalable and user-friendly tool for maximum likelihood phylogenetic inference. Bioinformatics.

[42] Sievers F, Wilm A, Dineen D, Gibson TJ, Karplus K, Li W, Lopez R, McWilliam H, Remmert M, Söding J (2011). Fast, scalable generation of high-quality protein multiple sequence alignments using Clustal Omega. Mol Syst Biol.

[43] Schubert M, Lindgreen S, Orlando L (2016). AdapterRemoval v2: rapid adapter trimming, identification, and read merging. BMC Res Notes.

[44] Jónsson H, Ginolhac A, Schubert M, Johnson PLF, Orlando L (2013). mapDamage2.0: fast approximate Bayesian estimates of ancient DNA damage parameters. Bioinformatics.

[45] Revell LJ (2012). phytools: an R package for phylogenetic comparative biology (and other things): phytools: R package. Methods Ecol Evol.

[46] Li H, Durbin R (2011). Inference of human population history from individual whole-genome sequences. Nature.

[47] Purcell S, Neale B, Todd-Brown K, Thomas L, Ferreira MAR, Bender D, Maller J, Sklar P, de Bakker PIW, Daly MJ, Sham PC (2007). PLINK: a tool set for whole-genome association and population-based linkage analyses. Am J Hum Genet.

[48] Ravinet M, Meier J (2020). Speciation & population genomics: a how-to-guide.

[49] Broad Institute (2021). Picard.

[50] Li H, Durbin R (2009). Fast and accurate short read alignment with Burrows-Wheeler transform. Bioinformatics.

[51] Ronquist F, Huelsenbeck JP (2003). MrBayes 3: Bayesian phylogenetic inference under mixed models. Bioinformatics.

[52] Ramsey CB (1995). Radiocarbon calibration and analysis of stratigraphy: the OxCal program. Radiocarbon.

[53] Lanfear R, Frandsen PB, Wright AM, Senfeld T, Calcott B (2017). PartitionFinder 2: new methods for selecting partitioned models of evolution for molecular and morphological phylogenetic analyses. Mol Biol Evol.

[54] McKenna A, Hanna M, Banks E, Sivachenko A, Cibulskis K, Kernytsky A, Garimella K, Altshuler D, Gabriel S, Daly M, DePristo MA (2010). The Genome Analysis Toolkit: a MapReduce framework for analyzing next-generation DNA sequencing data. Genome Res.

[55] Rice P, Longden I, Bleasby A (2000). EMBOSS: the European Molecular Biology Open Software Suite. Trends Genet.

[56] Kolde R (2015). pheatmap: Pretty Heatmaps.

[57] Quinlan AR, Hall IM (2010). BEDTools: a flexible suite of utilities for comparing genomic features. Bioinformatics.

[58] Page AJ, Taylor B, Delaney AJ, Soares J, Seemann T, Keane JA, Harris SR (2016). *SNP-sites*: rapid efficient extraction of SNPs from multi-FASTA alignments. Microb Genom.

[59] Yang Z (2007). PAML 4: phylogenetic analysis by maximum likelihood. Mol Biol Evol.

[60] Green RE, Malaspinas A-S, Krause J, Briggs AW, Johnson PLF, Uhler C, Meyer M, Good JM, Maricic T, Stenzel U (2008). A complete Neandertal mitochondrial genome sequence determined by high-throughput sequencing. Cell.

[61] Meyer M, Kircher M (2010). Illumina sequencing library preparation for highly multiplexed target capture and sequencing. Cold Spring Harb Protoc.

[62] Srivastava A, Kumar Sarsani V, Fiddes I, Sheehan SM, Seger RL, Barter ME, Neptune-Bear S, Lindqvist C, Korstanje R (2019). Genome assembly and gene expression in the American black bear provides new insights into the renal response to hibernation. DNA Res.

[63] Miller W, Schuster SC, Welch AJ, Ratan A, Bedoya-Reina OC, Zhao F, Kim HL, Burhans RC, Drautz DI, Wittekindt NE (2012). Polar and brown bear genomes reveal ancient admixture and demographic footprints of past climate change. Proc Natl Acad Sci USA.

[64] Li H (2021). Bcftools.

[65] Reimer PJ, Austin WEN, Bard E, Bayliss A, Blackwell PG, Ramsey CB, Butzin M, Cheng H, Lawrence Edwards R, Friedrich M (2020). The IntCal20 Northern Hemisphere radiocarbon age calibration curve (0–55 cal kBP). Radiocarbon.

[66] Boessenkool S, Hanghøj K, Nistelberger HM, Der Sarkissian C, Gondek AT, Orlando L, Barrett JH, Star B (2017). Combining bleach and mild predigestion improves ancient DNA recovery from bones. Mol Ecol Resour.

[67] Dabney J, Knapp M, Glocke I, Gansauge M-T, Weihmann A, Nickel B, Valdiosera C, García N, Pääbo S, Arsuaga J-L, Meyer M (2013). Complete mitochondrial genome sequence of a Middle Pleistocene cave bear reconstructed from ultrashort DNA fragments. Proc Natl Acad Sci USA.

[68] Vilstrup JT, Seguin-Orlando A, Stiller M, Ginolhac A, Raghavan M, Nielsen SCA, Weinstock J, Froese D, Vasiliev SK, Ovodov ND (2013). Mitochondrial phylogenomics of modern and ancient equids. PLoS ONE.

[69] Kapp JD, Green RE, Shapiro B (2021). A fast and efficient single-stranded genomic library preparation method optimized for ancient DNA. J Hered.

[70] Bolger AM, Lohse M, Usadel B (2014). Trimmomatic: a flexible trimmer for Illumina sequence data. Bioinformatics.

[71] Saremi NF, Oppenheimer J, Vollmers C, O’Connell B, Milne SA, Byrne A, Yu K, Ryder OA, Green RE, Shapiro B (2021). An annotated draft genome for the Andean bear, Tremarctos ornatus. J Hered.

[72] Kumar V, Lammers F, Bidon T, Pfenninger M, Kolter L, Nilsson MA, Janke A (2017). The evolutionary history of bears is characterized by gene flow across species. Sci Rep.

[73] Li R, Fan W, Tian G, Zhu H, He L, Cai J, Huang Q, Cai Q, Li B, Bai Y (2010). The sequence and de novo assembly of the giant panda genome. Nature.

[74] Liu S, Lorenzen ED, Fumagalli M, Li B, Harris K, Xiong Z, Zhou L, Korneliussen TS, Somel M, Babbitt C (2014). Population genomics reveal recent speciation and rapid evolutionary adaptation in polar bears. Cell.

[75] Mitchell KJ, Bray SC, Bover P, Soibelzon L, Schubert BW, Prevosti F, Prieto A, Martin F, Austin JJ, Cooper A (2016). Ancient mitochondrial DNA reveals convergent evolution of giant short-faced bears (Tremarctinae) in North and South America. Biol Lett.

[76] Kearse M, Moir R, Wilson A, Stones-Havas S, Cheung M, Sturrock S, Buxton S, Cooper A, Markowitz S, Duran C (2012). Geneious Basic: an integrated and extendable desktop software platform for the organization and analysis of sequence data. Bioinformatics.

[77] Stamatakis A (2014). RAxML version 8: a tool for phylogenetic analysis and post-analysis of large phylogenies. Bioinformatics.

