# Supplementary material for: Environmental genomics of Late Pleistocene black bears and giant short-faced bears
Source: Curr Biol. Author manuscript; Available in PMC 2025 Mar 2. (PMC7617452; doi:10.1016/j.cub.2021.04.027)
Supplement: Figure S1, Figure S2, Figure S2, Figure S4 [file EMS203357-supplement-Figure_S1__Figure_S2__Figure_S2__Figure_S4.pdf]

Current Biology, Volume *31*

Supplemental Information

Environmental genomics of Late Pleistocene black bears and giant short-faced bears

Mikkel Winther Pedersen, Bianca De Sanctis, Nedda F. Saremi, Martin Sikora, Emily E. Puckett, Zhenquan Gu, Katherine L. Moon, Joshua D. Kapp, Lasse Vinner, Zaruhi Vardanyan, Ciprian F. Ardelean, Joaquin Arroyo-Cabrales, James A. Cahill, Peter D. Heintzman, Grant Zazula, Ross D.E. MacPhee, Beth Shapiro, Richard Durbin, and Eske Willerslev

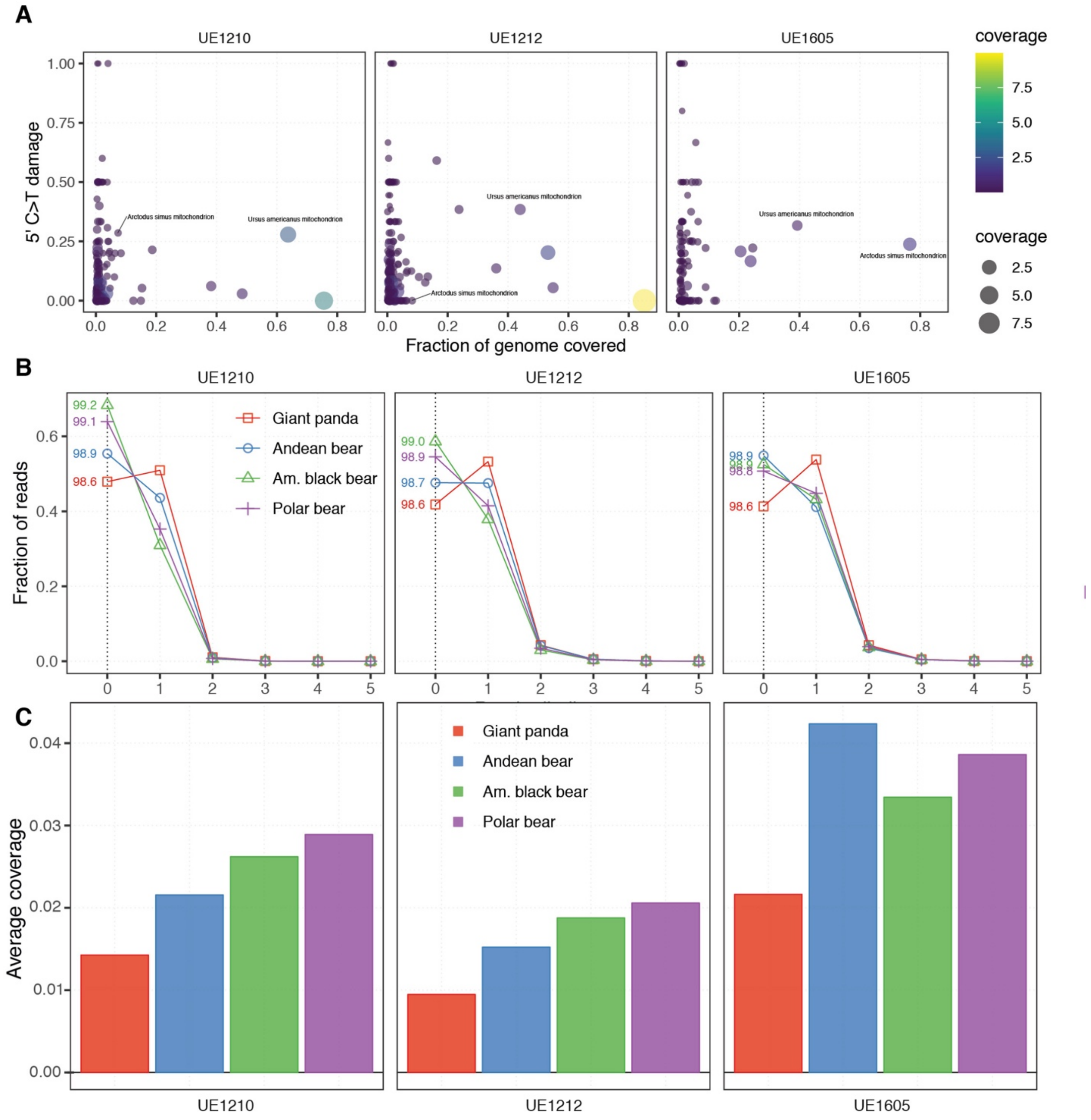

Figure S1. Exploratory mapping results. Related to Figure 1, 3 and 4, and Table S1. A. Mitochondrial coverage and C>T frequencies on 1st position against the RefSeq mitochondrial database. B. Read edit distances against four full reference genomes of bears. Values on the left indicate the average nucleotide identity of the all mapped reads to the respective reference. C. Barplot of average genome coverage, we find the average coverage similar between polar bear and American black bear, but the reads are closer to American black bear.

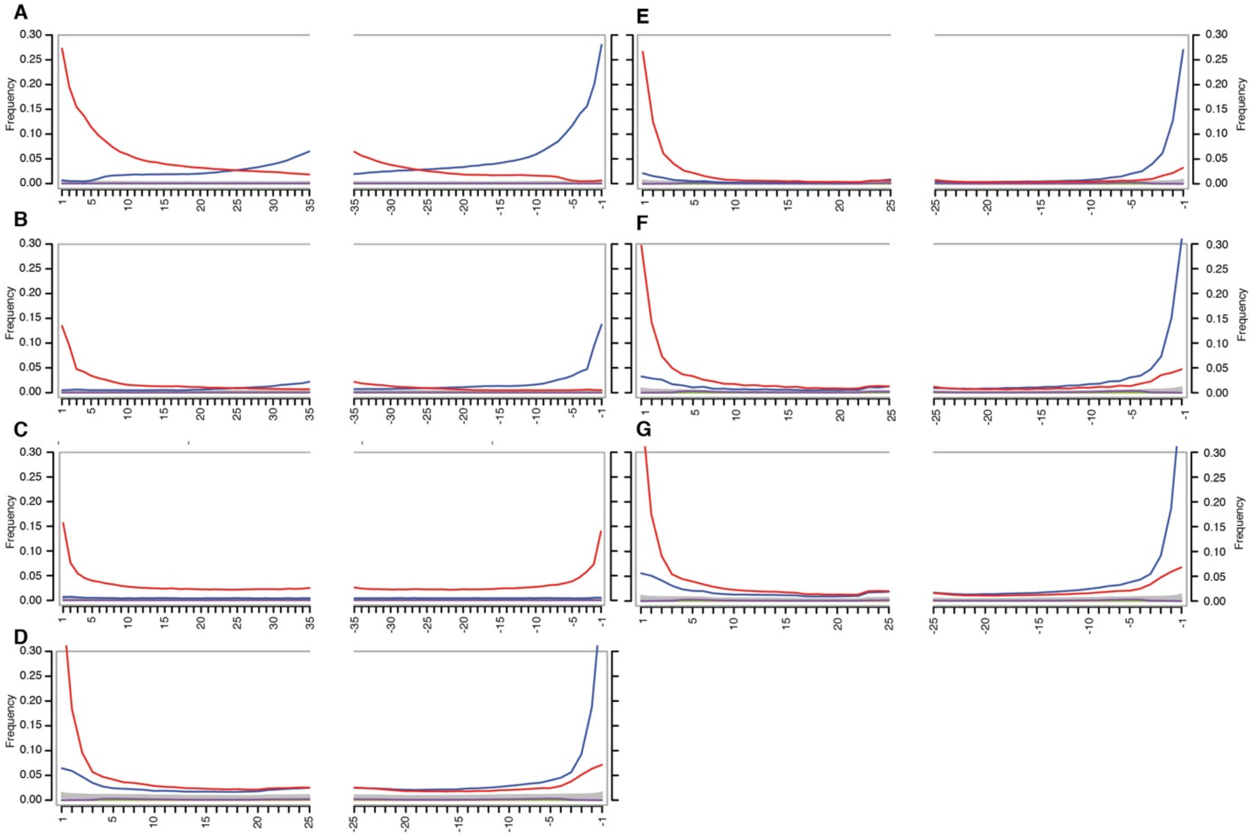

Figure S2. Ancient DNA authentication using substitution frequencies. Related to Figure 1, 3, 4 and Table S1. Lines show the position specific substitutions of the 25-35 first base pairs from both the 5' and the 3' end. The following colors represent C to T substitutions (red), G to A substitutions (blue), all other substitutions (grey), soft-clipped bases (orange), deletions relative to the reference (green) and insertions relative to the reference (purple). **A-C.** show the Yukon short-faced bear fossils substitutions when aligned to the Andean bear genome, **A.** YG 24.1 **B.** YG 76.4, **C.** YG 546.562. While **D** shows the substitutions found for UE1605 when aligning the environmental genomes to Andean bear genome, and **E-G.** show the substitutions found when aligning the environmental genomes to the American black bear genome (**E.** UE1210, **F.** UE1212, **G.** UE1605).

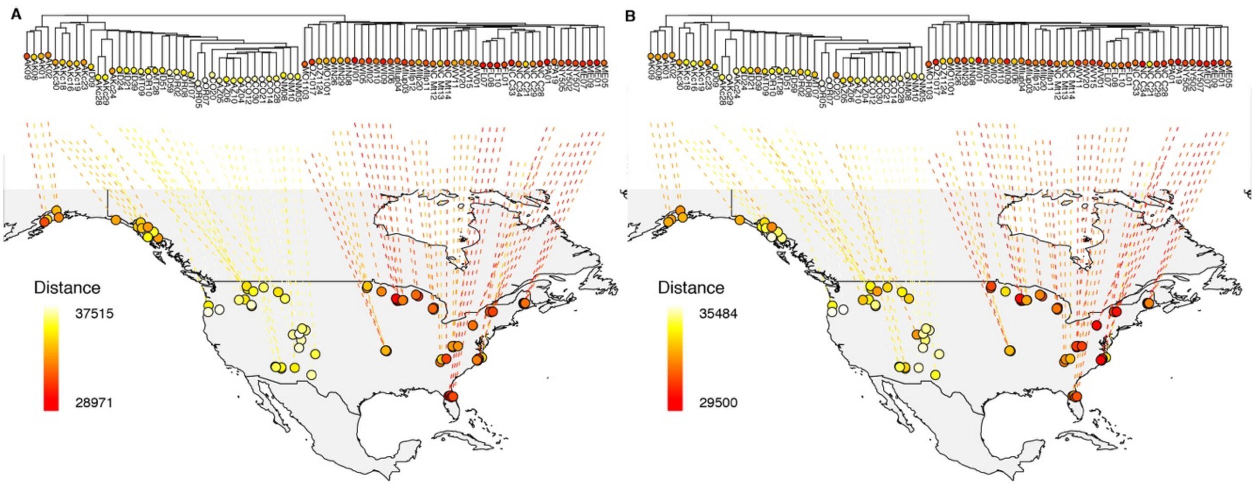

Figure S3. Genetic Hamming distance of ancient Mexican black bear samples. Related to Figure 1 and 2, and Table S2. Samples UE1210 and UE1605 (shown in (A) and (B) respectively), mapped to a colour scale and plotted on a *phylomap* using a neighbour-joining tree of the modern samples.

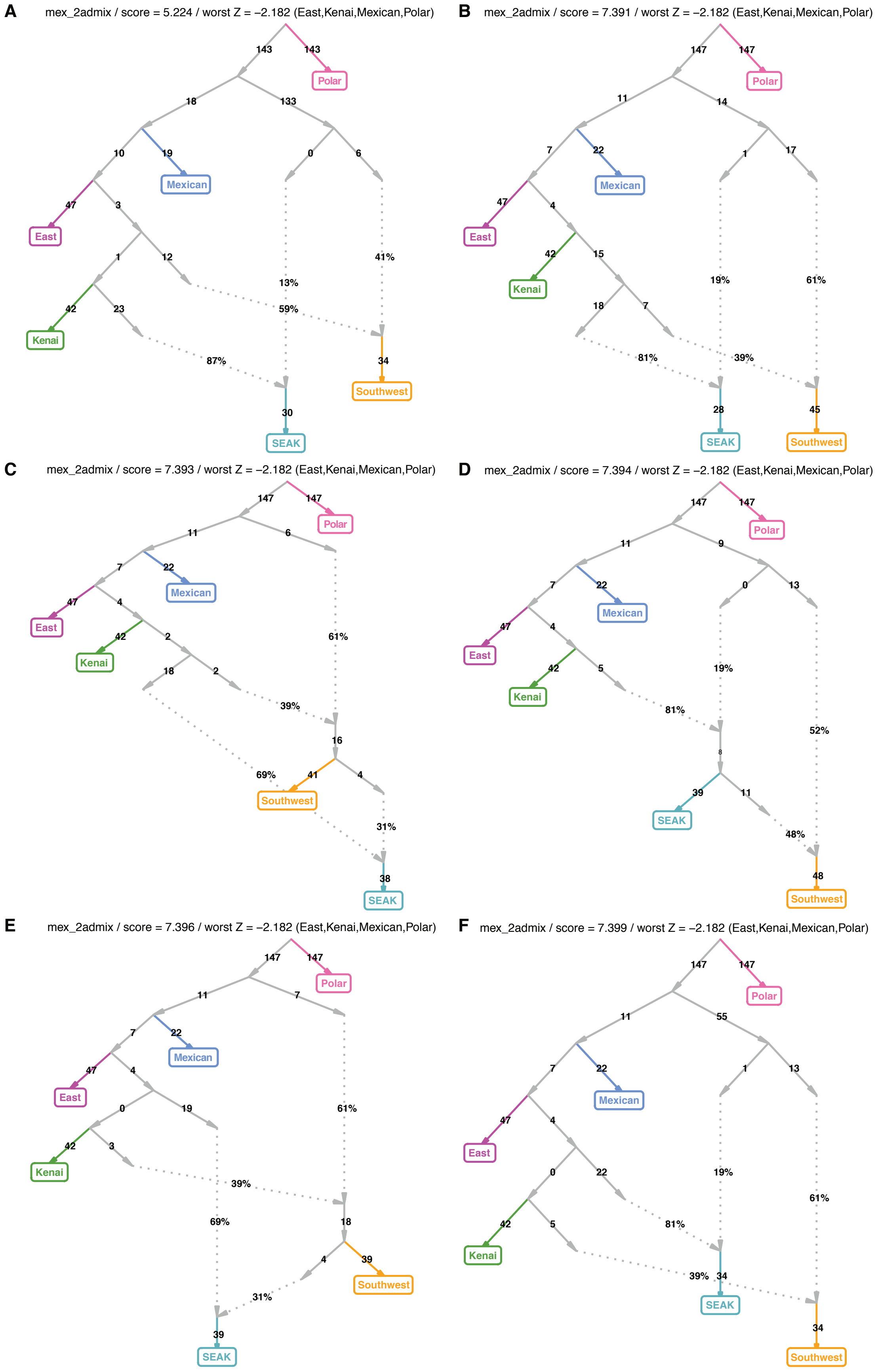

A
mex_2admix / score = 5.224 / worst Z = −2.182 (East,Kenai,Mexican,Polar)
Polar
Mexican
East
Kenai
SEAK
Southwest
B
mex_2admix / score = 7.391 / worst Z = −2.182 (East,Kenai,Mexican,Polar)
Polar
Mexican
East
Kenai
SEAK
Southwest
C
mex_2admix / score = 7.393 / worst Z = −2.182 (East,Kenai,Mexican,Polar)
Polar
Mexican
East
Kenai
Southwest
SEAK
D
mex_2admix / score = 7.394 / worst Z = −2.182 (East,Kenai,Mexican,Polar)
Polar
Mexican
East
Kenai
SEAK
Southwest
E
mex_2admix / score = 7.396 / worst Z = −2.182 (East,Kenai,Mexican,Polar)
Polar
Mexican
East
Kenai
Southwest
SEAK
F
mex_2admix / score = 7.399 / worst Z = −2.182 (East,Kenai,Mexican,Polar)
Polar
Mexican
East
Kenai
SEAK
Southwest

Figure S4. Best black bear admixture graphs. Related to Figure 1 and 2, and Table S2. Our admixture analysis using *admixtools* produced seven best black bear admixture graphs. The best of these is shown in Figure 1D, and the remaining six are shown here (a lower score is better). Each of these has the same worst excess f4 residual z-score of -2.182 for the configuration (East,Kenai;Mexican,Polar), but scores slightly differently. All seven graphs share many common characteristics, as noted in the STAR Methods.
